# Supplementary material for: Genomic insights into the host specific adaptation of the Pneumocystis genus
Source: Commun Biol. 2021 Mar 8;4:305. doi: 10.1038/s42003-021-01799-7 (PMC7940399; doi:10.1038/s42003-021-01799-7)
Supplement: Supplementary file 1 — Supplementary Information [file 42003_2021_1799_MOESM1_ESM.pdf]

Supplementary Materials for

Genomic insights into the host specific adaptation of the *Pneumocystis* genus

Ousmane H. Cissé, Liang Ma, John P. Dekker, Pavel P. Khil, Jung-Ho Youn, Jason M. Brenchley, Robert Blair, Bapi Pahar, Magali Chabé, Koen K.A. Van Rompay, Rebekah Keesler, Antti Sukura, Vanessa Hirsch, Geetha Kutty, Yueqin Liu, Li Peng, Jie Chen, Jun Song, Christiane Weissenbacher-Lang, Jie Xu, Nathan S. Upham, Jason E. Stajich, Christina A. Cuomo, Melanie T. Cushion, Joseph A. Kovacs

Correspondence to: ousmane.cisse@nih.gov

This PDF file includes:

Supplementary Methods
Supplementary Notes 1 and 2
Supplementary Figures 1 to 11
Supplementary Tables 1 to 5
Supplementary References

Supplementary Methods:

P. macacae genome sequencing: Host specific Illumina reads were removed by mapping against Rhesus macaque genome (NCBI accession number GCF_000772875.2_Mmul_8.0.1) using Bowtie2 [1] with the --sensitive-local option. Unmapped reads were merged and analyzed using GenomeScope [2] to estimate whether coverage was sufficient to successfully recover the genome. Genome assembly was performed using Spades v3.11.1 [3] and resulting scaffolds were aligned to the macaque genome using BLAT [4] with default parameters to remove residual contaminant contigs. Bacterial contaminants were removed by comparing the remaining scaffolds to NCBI nr database using BLASTx [5]. Remaining scaffolds were compared to a database containing the published genomes of *P. jirovecii*, *P. carnii* and *P. murina* (GCA_001477535.1, GCA_001477545.1 and GCF_000349005.1) using BLAT with a minimum score of 200 nucleotides and a minimal identity of 70%. Scaffolds without significant similarity with *Pneumocystis* genomes as determined by BLAT were compared to a custom database of fungal proteins using TBLASTn [5] with an e-value of 0.01 as threshold. Mitochondrial contigs were identified by BLASTn against published *Pneumocystis* mitogenomes and removed.

P. wakefieldiae genome sequencing: *P. wakefieldiae* specimens were obtained from five rats (*Rattus norvegicus*) including three brown Norway rats and two Long Evans breed rats. Rats were acquired from different commercial vendors and housed at the University of Cincinnati from September 1995 to September 2002. *Pneumocystis* pneumonia was induced by corticosteroids-induced immunosuppression. All rats were co-infected by both *P. wakefieldiae* (NCBI taxonomy ID: 38082) and *P. carinii* (ID:4754) (Supplementary Table 1). We sequenced

one genomic DNA preparation from each of the four rats and one sample of total RNA preparation from a single rat (Supplementary Table 2).

Raw Illumina HiSeq reads were filtered using trimmomatic [6] and aligned to the *Rattus norvegicus* genome (NCBI accession no. GCF_000001895.5) using Bowtie2 v.2.2.5 with the following parameters –very-fast –no-discordant –no-mixed. Unmapped reads were mapped against the *P. carinii* genome (NCBI accession # GCA_001477545.1) using Bowtie2 version 2.2.5 with the following parameters: -no-discordant -D 5 -R 1 -N 0 -L 32 -i S 0,2.50 –end-to-end. Reads that failed to map to Rat or *P. carinii* genomes were considered for initial genome assembly using Spades [3]. We initially attempted to generate an assembly per isolate. However, this strategy yielded highly fragmented assemblies indicating insufficient coverage per sample. Of note, the removal of presumed polymorphic major surface glycoproteins (*msg*) gene reads before assembly did not substantially improved the assembly continuity or completeness. Therefore, *msg*-related reads were not specifically removed before assembly in the subsequent steps.

Subsequently, filtered reads (i.e. after removing of high confidence rat and *P. carinii* reads from all samples) from all four rats were merged and assembled with Spades. This assembly process yielded 139,106 contigs totalizing 77 Mb, which were aligned to rat genome (GCF_000001895.5) using BLAT version 3.5 [4] with default parameters. Small sized scaffolds (< 500 bp) were filtered out because they appeared to be enriched in rodent specific repeats. This reduced the dataset to 10,630 contigs corresponding to 23 Mb.

To test whether our assembly truly captured *P. wakefieldiae* but not *P. carinii*, we predicted 7,918 gene models from the 10,630 scaffolds using AUGUSTUS version 3.2.1 [7] with built-in *Pneumocystis* gene models [8]. We then performed an all-vs-all search using reciprocal

BLASTp hit search with an e-value of 10^{-10} as threshold against a custom database containing the complete proteomes of *P. carinii* strain B80 (*n* = 3,646; NCBI accession no. GCF_001477545.1), *P. carinii* strain Ccin (*n* = 3,305; genome assembly from [9] but annotated in this study), *P. carinii* strain SE6 (*n* = 3,506; genome assembly from sample BALE6 [10] but annotated in this study) and *P. murina* (*n* = 3,838 proteins; GCF_000349005.1). Nucleotide sequence comparisons of seven homologous genes showed 4-7% divergence between *P. wakefieldiae* and *P. carinii* whereas only 0 – 0.8% divergence was observed within *P. carinii* species [11]. Whole genome alignment-based divergence estimates between strains *P. carinii* can reach 5% (Supplementary Table 4), although this estimate may be inflated by higher error rates in strains Ccin and SE6. We BLASTed all the predicted genes against the *P. carinii* and *P. murina* reference gene sets (e-value of 10^{-20} as cut off). Candidate homologs were extracted and global pairwise identities were computed using Needle from EMBOSS package [12]. Genes with at least 80% similarity with *P. carinii* homologs were extracted and analyzed. No gene was found with an identity > 80% with *P. murina*. For each gene, an orthologous group including sequences from *P. jirovecii*, *P. carinii* (strains B80, Ccin, SE6), *P. murina* and *S. pombe* when possible was constructed using reciprocal best BLASTp hit with an e-value of 10^{-10} as cut off. Multiple sequence alignments were generated using MAFFT [13] and phylogenetic inferences made with RAxML [14]. Based on phylogenetic placements and pairwise identities, 2,710 gene models located in 10,380 scaffolds were considered as *P. carinii* and removed. To remove residual non-coding *P. carinii* genomic segments, the remaining scaffolds (*n* = 250, size= 13,423,939 bp, GC=28.9%) were aligned to *P. carinii* strain B80 genome (GCA_001477545.1) with MegaBLAST [15] using the following parameters "-W 1000 -v 1 -b 1 -e 1e-150". A total of 20 scaffolds were flagged by MegaBLAST and discarded after manual inspection, which left 230

scaffolds (7,200,860 bp, GC% = 30.1). We ran AUGUSTUS on these 230 scaffolds and clustered to predictions with the reference proteomes of *P. jirovecii*, *P. carinii* and *P. murina*. We identified 674 one-to-one orthologs, which were concatenated and used to build a phylogenetic tree using RAxML. This tree unambiguously placed *P. wakefieldiae* as distinct species from *P. carinii* and *P. murina*. After this validation step, we performed a gap closure and reduction using Redundans [16], which reduce the number of scaffolds to 134 totalizing 7.1 Mb (GC%=30).

We annotated these 134 scaffolds with AUGUSTUS and compared gene models to four published gene sequences of *P. wakefieldiae* sequences from Uniprot: Q01706 for guanine nucleotide binding protein alpha subunit, O00053 for PrBiP, O94108 for heat shock protein 70B/SSB1 (Fragment) and Q12652 for TATA binding protein. The PrBiP and TATA binding protein sequences were fully recovered using reciprocal Best BLASTp hit with an e-value of 10^{-10} as cut off. The pairwise amino acid identities of our gene models with published PrBiP and TATA were 98.8% and 98.7%, respectively, whereas the identities with *P. carinii*, *P. murina* and *P. jirovecii* ranged from 82.8% to 96.5%. As part of the verification process, we performed a synteny analysis, comparing our assembly to the genome sequence of *P. carinii* and *P. murina* using SatsumaSynteny2 [17]. We found that the gene order in our assembly was clearly different than orthologs in *P. carinii* and *P. murina*, which suggests a different species. To further verify the identity of our genome assembly, we identified the rRNA operon using BLAT and compared to the published *P. wakefieldiae* rRNA operon (GenBank accession no. L27658). We also identified a genomic fragment containing the mating-type locus cloned by PCR and Sanger sequenced in this study. While the rRNA operon was only partially recovered in our assembly (416 nt), the recovered part of the rRNA showed 99.8% nucleotide identity with the published

sequence. The rRNA operon contains internal repeats which likely causes the assembly breakpoints. The fragment containing the mating-type locus (~15 kb) was fully recovered and exhibited 100% nucleotide identity. All internal gaps were closed by PCR and Sanger sequencing. The final genome assembly has 17 scaffolds with a total size of 7.3 Mb, which appears to be consistent with the results of karyotypic studies [11].

P. canis genome sequencing: We sequenced two *P. canis* DNA samples from two dogs from Austria and Finland (denoted as A and Ck, respectively; Supplementary Table 1). Since previous studies of the mitochondrial large subunit rRNA gene (mtLSU) have demonstrated the presence of two types of *P. canis* populations with a significant variability in the dog Ck [18], we analyzed the sequencing data from samples A and Ck separately. Two genome assemblies were recovered from the sample Ck (denoted as Ck1 and Ck2) and one assembly from the sample A. Due to the lack of reliable karyotype data, it remains unclear how many chromosomes there are for *P. canis*.

Prior to the genome assembly, the filtered reads were analyzed using GenomeScope [2] to determine if sufficient reads are available for full genome recovery. Reads were filtered using trimmomatic [6]. Host reads were removed by mapping to dog reference genome (NCBI accession no. GCF_000002285.3) with Bowtie2 [1]. In the dog Ck, preliminary assembly of these reads revealed the presence of exogenous DNA from the plant *Arabidopsis thaliana* and the fungus *Puccina graminis*. These reads were considered contaminants and removed by mapping reads against their respective genomes (GCF_000001735.3_TAIR10 and GCF_000149925.1). No contaminant was observed in reads from the dog A other than DNA from the host. After filtering, reads from each sample were assembled separately using Spades [3]. We BLASTed the resulting scaffolds against UniProt bacterial database (available at ftp.uniprot.org/databases/uniprot) for

bacterial contamination using BLASTx [5] with an e-value of 10^{-15}. Residual dog contaminants (mostly microsatellites) were detected by screening against a database of 96 *Canis familiaris* clone CSac3 satellite sequences downloaded from NCBI. Redundant and heterologous scaffolds were identified using Redundans [16]. Haplotype analyses were performed using custom Perl scripts, which allow the identification of two populations.

P. oryctolagi genome sequencing: organisms were isolated from three rabbits collected in France and a single Interleukin-2 receptor-ɣ knockout rabbit with a severe combined immunodeficiency, from Michigan [19] (Supplementary Table 1). We sequenced a single genomic DNA specimen for each rabbit. The proportion of *Pneumocystis* DNA relative to host DNA was relatively low (< 5%; Supplementary Table 2). Filtered reads from all four samples were combined to generate a consensus genome assembly.

Illumina reads from the host were removed by mapping against rabbit genome (NCBI accession no. GCF_000003625.3_OryCun2.0) using Bowtie2 with the --sensitive-local option. Separate assembly of unmapped reads from each *P. oryctolagi* isolate resulted in a highly fragmented assemblies (for example, a total of 3.7 Mb in 2,645 contigs for the isolate RAB_F). Therefore, we pooled unmapped reads from all isolates and assembled them with Spades v3.11.1 [3]. Initial assembly provided 1,544,250 contigs totalizing 877 Mb. A second round of filtering was performed comparing all contigs to rabbit genome using BLAT. After removal of host contigs, the remaining scaffolds were compared to a database containing the published genomes of *P. jirovecii*, *P. carinii* and *P. murina* (NCBI accession nos. GCA_001477535.1, GCA_001477545.1, GCF_000349005.1) using BLAT with a minimum score of 200 and a minimal identity of 70%. Scaffolds with no significant similarity with *Pneumocystis* genomes

were compared to a custom database of fungal proteins using TBLASTn with an e-value of 0.01 as threshold. A total of 321 contigs totalizing 7.2 Mb passed that filtering step. To test if heterologous scaffolds were present in the assembly, we analyzed the 321 contigs using Redundans. Only 18 heterologous contigs were detected, of which 17 are smaller than 500 bp in size. The reduced assembly produced by Redundans pipeline was not used in subsequent analyses. We used "chromoAssemble" module of Satsuma2 [17] and *P. jirovecii* strain RU7 genome as reference to guide the synteny-based scaffolding. Genome annotation was performed using Funannotate (https://zenodo.org/record/2604804). The annotated heat shock protein 70 gene displayed 96% of nucleotide identity with the published sequence (DQ435616 [20]), which indicates some strain variation within *P. oryctolagi* populations.

Pneumocystis mitochondrial genomes assembly and annotation: Mitogenome reads were retrieved from Illumina HiSeq reads by mapping against published mitogenomes of *P. jirovecii, P. carinii* and *P. murina* using SeqMan NGen (version 14.1.0.118, DNASTAR, Madison, WI) under default conditions except for reducing the minimum match percentage to 70%. Retrieved reads were *de novo* assembled using SeqMan NGen under default conditions except for increasing the minimum match percentage to 97%. The resulting contigs were assembled using Sequencher (version 5.4.6; Gene Codes Co., MI) and compared to published *Pneumocystis* mitogenomes. Gaps were filled by multiple rounds of alignment of merged contigs to Illumina raw reads. For *P. wakefieldiae*, retrieved mitogenome reads were first aligned to the *P. carinii* mitogenome (GenBank accession number JX499145) with a minimum match percentage of 97%. Unaligned reads were *de novo* assembled using SeqMan NGen. All final assemblies were re-aligned to Illumina raw reads to check for any potential errors or inconsistencies. In addition, the

final assembly of *P. wakefieldiae* and *P. macacae* mitogenome was amplified as 6-8 overlapping fragments from genomic DNA by PCR; selected regions from each PCR product were sequenced directly by Sanger Sequencing.Mitogenome annotation was performed using the MFannot tool with genetic code 3 for yeast mitogenome (http://megasun.bch.umontreal.ca/cgi-bin/mfannot/mfannotInterface.pl). Transfer RNAs were further evaluated using tRNAscan-SE [21]. All annotated genes were reviewed and compared with the homologs in *P. jirovecii, P. murina* and *P. carinii* mitogenomes.

Supplementary Text:

Note 1: Population genomics analysis

Analysis of *P. jirovecii*, *P. macacae* and *P. oryctolagi*:

Coevolution of pathogenic species with their hosts leaves genomic footprints which can, if the divergence times are recent enough, be identified and characterized. These regions which are often referred as "genomic islands of differentiation" can reveal insights into the interaction of the pathogen with its host. We used genome scans to identify these regions with significant divergence relative to average genomic divergence. We found that the *Pneumocystis* genomes display a high level of genetic differentiation and are mostly evolving under weak purifying selection or neutral evolution. Genomic islands could not be identified because they have been eroded over the large evolutionary timescale that separates different *Pneumocystis* species. Specifically, to understand the genomic divergence landscape of *Pneumocystis* populations, we performed genome-wide differentiation tests (F_{ST}, relative population divergence) and nucleotide diversity (π). We used a trained version LAST [22] to account for interspecies divergence during read mapping and ANGSD [23] to derived genotype likelihoods instead of genotypes. To analyze

the genomic landscape of *P. jirovecii*, we aligned Illumina reads from *P. macacae*, *P. oryctolagi* and *P. carinii* to the *P. jirovecii* reference genome strain RU7. Figure S3a illustrates the distribution of each isolate according to cluster distance. Hierarchical clustering unambiguously separates isolates from different species and indicates that the majority of genetic variation within this data set consists of inter-species fixed differences. F_{ST} scans reveal a differentiation between *P. jirovecii* and *P. macacae* populations and an even greater divergence with *P. oryctolagi*. In both comparisons, the genome-wide distributions of F_{ST} are unimodal with highest peaks at 0.98 and 0.93 (Supplementary Figure 3b). In fact, 71.9% of the total 16,825 windows of 5 kb are highly differentiated ($F_{ST} > 0.8$) in *P. jirovecii-P. macacae* comparison, and this number reaches 90.2% in *P. jirovecii-P. oryctolagi* comparison. Regions of $F_{ST} < 0.5$ are negligible 0.5% and 0.0%, respectively. Inspection of genomic regions with low F_{ST} reveals that they are significantly enriched in regions encoding highly polymorphic Msg, which suggests that the low F_{ST} values are caused by local increases in genetic diversity.

Analysis for the trio *P. murina*, *P. carinii* and *P. wakefieldiae*: We aligned reads from *P. wakefieldiae*, *P. carinii, P. murina* and *P. jirovecii* to the *P. murina* reference genome (raw reads statistics are presented in Supplementary Table 2). Our interspecies mapping strategy was designed to account for interspecies divergence of ~20% using LAST. We performed a clustering analysis of the SNP genotype data. Of a total of 7,390,170 sites analyzed, 2,939,752 sites were retained after filtering). Figure 3a illustrates the distribution of each isolate according to distances. Hierarchical clustering cleanly separates samples from different species and indicates the majority of genetic variation within this data set consists of inter-species fixed differences. This allows computation of F_{ST} values comparing *P. murina* population ($n = 12$) to *P. carinii* ($n = 7$) and *P. wakefieldiae* ($n = 5$). F_{ST} genome scans reveal a significant population

differentiation between *P. murina* and other species. In the comparison between *P. murina* and *P. wakefieldiae* populations, the genome-wide distribution of F_{ST} is unimodal with its highest peak at 0.99, which indicates that most of the *P. murina* genome is fully differentiated relative to *P. wakefieldiae*. In contrast, the comparison between *P. murina* and *P. carinii*, F_{ST} distribution is bimodal with two peaks at 0.9 and 0.99. A total of 86.3% and 93.7% of the 14,912 windows (5 kb) have an F_{ST} value > 0.8 in *P. murina-P. carinii* and *P. murina-P. wakefieldiae* comparisons, respectively. Genomic regions with F_{ST} < 0.5 represent 0.2% and 0.06%, respectively. Inspection of genomic regions with low differentiation (F_{ST} < 0.5) reveals a high incidence of SNPs, which suggests that low F_{ST} is caused by local increase in genetic diversity.

Note 2: Metabolic pathways.

Amino acids biosynthesis: The reduction of amino acid metabolism observed in *P. jirovecii*, *P. carinii* and *P. murina* [24] is also observed in *P. macacae*, *P. oryctolagi*, *P. canis* and *P. wakefieldiae* in this study. That is ~80% of the genes involved in amino acid biosynthesis in yeast are missing whereas most of their homologs are present in other Taphrinomycotina, which indicates that the loss of amino acid biosynthetic ability occurred in the LCA of *Pneumocystis*. All *Pneumocystis* have impaired capacity for assimilation of inorganic nitrogen and sulfur. Therefore, none of the 20 standard amino acids can be synthetized *de novo* although a few can be derived from others. There is only one amino acid transporter (*ptr2*) gene which is also conserved in all sequenced species. There is no amino acid permease in any sequenced *Pneumocystis* species. Transporters are significantly depleted.

It is believed that *Pneumocystis* scavenges amino acids from its host largely through mitochondrion and vacuolar associated amino-acid transporters. The unique amino acid

transporter gene (*avt3*) is conserved in *P. jirovecii*, *P. macacae* and *P. oryctolagi*, but lost in the other 4 species sequenced. However, the directionality of AVT3 protein is unclear because it is usually used to import compounds from cytoplasm to help regulate intracellular amino acid levels [25]. The polyamine transporter AQR1, which is believed to be essential for *Pneumocystis* to scavenge polyamine [26], is only present in *P. jirovecii*, *P. macacae* and *P. canis*. Similarly, AQR1 is an internal membrane protein involved in the excretion of excess of amino acids [27], thus it is unclear whether the AQR1 transporter can also be used to import amino acids from the extracellular environment. These differences suggest that AVT3 and AQR1 are not essential to *Pneumocystis* survival and the loss may be due to a stochastic event. Apart from the few genes mentioned above, we found no significant differences in metabolic pathways among *Pneumocystis* species (Wilcoxon signed-rank test, p-value = 0.7).

Serine/glycine biosynthesis: The serine/glycine biosynthesis pathway is probably nonfunctional in *Pneumocystis* because the key enzyme isocitrate lyase 1 as well as half of the 13 genes in this pathway in yeast are absent in all *Pneumocystis*. The glycine cleavage system (glycine decarboxylase complex), which catalyzes the degradation of glycine is likely functional because nearly all species possess three copies of glycine decarboxylases (*gcv1*, *gcv2*, *gcv3*). The only exception is *P. oryctolagi*, which lacks two of the three glycine decarboxylases and thus is probably unable to recycle glycine. *P. canis* genome lacks the glycine hydroxymethyltransferase gene (*shm1*), which is involved in the interconversion of serine and glycine.

Sulfur metabolism: Sulfur metabolism and synthesis of precursors for sulfur containing amino acids (methionine, cysteine, homocysteine, and taurine) is probably deficient because 13 of 22

genes of the pathway are lost in *Pneumocystis*. Key enzymes such as bifunctional cysteine synthase or cystathionine gamma-synthase are missing in all *Pneumocystis*. The cystathionine gamma lyase (*cys3*) gene, which is involved in the step 2 of synthesis of L-cysteine from L-homocysteine and L-serine, is missing in *P. oryctolagi* and *P. canis*. *P. oryctolagi* lacks the cystathionine beta-synthase (*cys4*) gene, which is involved in the step 1 of synthesis of L-cysteine from L-homocysteine and L-serine. These results suggest that these compounds are acquired from the host.

Carbohydrate metabolism: All necessary genes for uptake and catabolism of glucose via glycolysis and the tricarboxylic acid (TCA) cycle are present in *Pneumocystis*. Genes involved in the conversation of fructose and mannose to glucose, and the synthesize of glycogen and trehalose are present in all species. Key enzymes that convert galactose and sucrose are missing (galactokinase and beta-fructofuranosidase) are missing in *Pneumocystis* while genes for galactose metabolism are conserved in other Taphrinomycotina, which indicates that this pathway is lost specifically in the *Pneumocystis* branch. Additional losses include two enzymes for glyoxylation, one key enzyme for gluconeogenesis and all enzymes for pyruvate fermentation. These findings suggest that the reliance on glucose for energy production is a general feature for *Pneumocystis* species as suggested in our previous study [28].

Fatty acid metabolism: As observed before [28], most of the genes involved in the fatty acid beta-oxidation are missing, which further supports that hypothesis that fatty acids are not an energy source for *Pneumocystis*. *Pneumocystis* organisms are thought to rely on glycerol for maintaining osmotic balance. Although the biosynthetic machinery of glycerol from glycerone-phosphate or

monoacylglycerol is lost in all species, glycerol uptake and export proteins GUP1 and FPS1 are conserved in all species except *P. macacae* and *P. canis* which have lost the *fps1* gene. Interestingly, *P. oryctolagi* and *P. canis* genomes have a single copy of *dga1* gene (coding for the 2-acylglycerol O-acyltransferase 2; EC: 2.3.1.22), which is involved in glycerolipid synthesis. The *dga1*gene is absent in other *Pneumocystis* species.

Pantothenate *de novo* biosynthesis: the pathway is lost in all *Pneumocystis* species, whereas the ability to convert the pantothenate to the coenzyme A (CoA) is conserved in all *Pneumocystis*. Given that the pantothenate specific transporter gene (*Fen2*) is also lost, we have previously suggested that *Pneumocystis* uses the CoA transporter LEU5 to scavenge the pantothenate or its downstream metabolites from the hosts [28].

Sterol metabolism: Lipid metabolism in *Pneumocystis* is comparable among species. Our previous study suggested that *P. jirovecii*, *P. carinii* and *P. murina* can synthetize fecosterol and episterol, but are unable to convert them to ergosterol because of the absence of genes coding for two late stage enzymes ERG3 and ERG5 in the two latter species [28]. Moreover, both human and rodent *Pneumocystis* lost the gene for the key enzyme Dhcr24 (24-dehydrocholesterol reductase; EC 1.3.1.72) required for cholesterol biosynthesis [24]. *P. macacae*, *P. oryctolagi* and *P. canis* genomes encode homologs of *erg3* and *dhcr7* whereas *P. wakefieldiae* lacks both of them. All species lack the key enzyme Dhcr24 required for cholesterol biosynthesis. This suggests that none of these species can synthesize cholesterol but presumably have the ability to scavenge it from the host though the genes involved in cholesterol acquisition have not been identified.

Cofactor metabolism: The riboflavin biosynthetic pathway is conserved in all *Pneumocystis* with the exception of *P. canis* which lacks the *rib7* gene (2,5-diamino-6-ribosylamino-4(3H)-pyrimidinone 5'-phosphate reductase), which encodes a key enzyme required to catalyze an early step in riboflavin biosynthesis [29]. The sequenced species also lack genes for *de novo* synthesis of vitamins B1 and H. Genes encoding siderophores are also largely missing. The potential plasma membrane transporter for each of these cofactors is however highly conserved in all Taphrinomycotina including *Pneumocystis*. The nicotinamide adenine dinucleotide (NAD) *de novo* synthesis pathway is severely truncated in *P. jirovecii* with loss of 6 out 9 genes. *P. macacae* also presents a reduction in this pathway, although less severe with 5/9 genes lost (the addition compared to *P. jirovecii* is the *bna6* gene (nicotinate-nucleotide diphosphorylase carboxylating). In contrast *P. oryctolagi* has 8/9 genes, with only the *bna2* gene (indoleamine 2,3-dioxygenase) missing. The *bna2* gene is also lost in *Schizosaccharomyces and Protomyces* while the *bna6* gene is lost in *Schizosaccharomyces, Protomyces* and *Neolecta*. The BNA2 enzyme catalyzes the first step of tryptophan catabolism in order to supply *de novo* nicotinamide adenine dinucleotide (NAD) via the kynurenine. BNA6 is involved in the catabolism of quinolinic acid. *P. canis*, *P. carinii*, *P. murina* and *P. wakefieldiae* have all the enzymes required for NAD *de novo* synthesis. The NAD salvage pathway including the nicotinic acid transporter is conserved in all these species. Some of these genes are lost in *Schizosaccharomyces* and *Protomyces* i.e. *bna1* (encoding the HAD1 3-hydroxyanthranilate 3,4-dioxygenase) and *bna2*. This might indicate that the catabolism of tryptophan and quinolinic acid are not used by *P. jirovecii*, *P. macacae* and *P. oryctolagi*.

B6 metabolism: Vitamin B6, which is an essential metabolite involved in defense against cellular oxidative stress, is synthesized by the DXP-independent pathway [30]. The pathway involves two

enzymes: PDX1 (Pyridoxal 5'-phosphate synthase) and PDX2. We found that *pdx1* gene is conserved in all except primate *Pneumocystis* species (*P. jirovecii* and *P. macacae*) whereas *pdx2* gene is conserved only in rodent-infecting species (*P. carinii*, *P. murina* and *P. wakefieldiae*). The B6 salvage pathway involves three enzymes: BUD16 (putative pyridoxal kinase), PDX3 (pyridoxamine-phosphate oxidase) and TPN1 (plasma membrane pyridoxine transporter). We identified orthologs of *bud16* gene in all *Pneumocystis* genomes and *pdx3* in all but *P. oryctolagi*, while *tpn1* is absent in all *Pneumocystis*. This uneven gene distribution pattern suggests that the *de novo* synthetic pathway for vitamin B6 may be functional only in rodent *Pneumocystis*, and that other *Pneumocystis* might have developed alternative strategies to generate B6, including scavenging from the host intermediate metabolites.

Figure S1. The maximum clade credibility tree of *Pneumocystis* summarized by TreeAnnotator and plotted against stratigraphy using the strap package in R. The internal nodes of the tree are indicated with circles, where the circles mark nodes with posterior probability: plain black > 0.95, grey > 0.75.

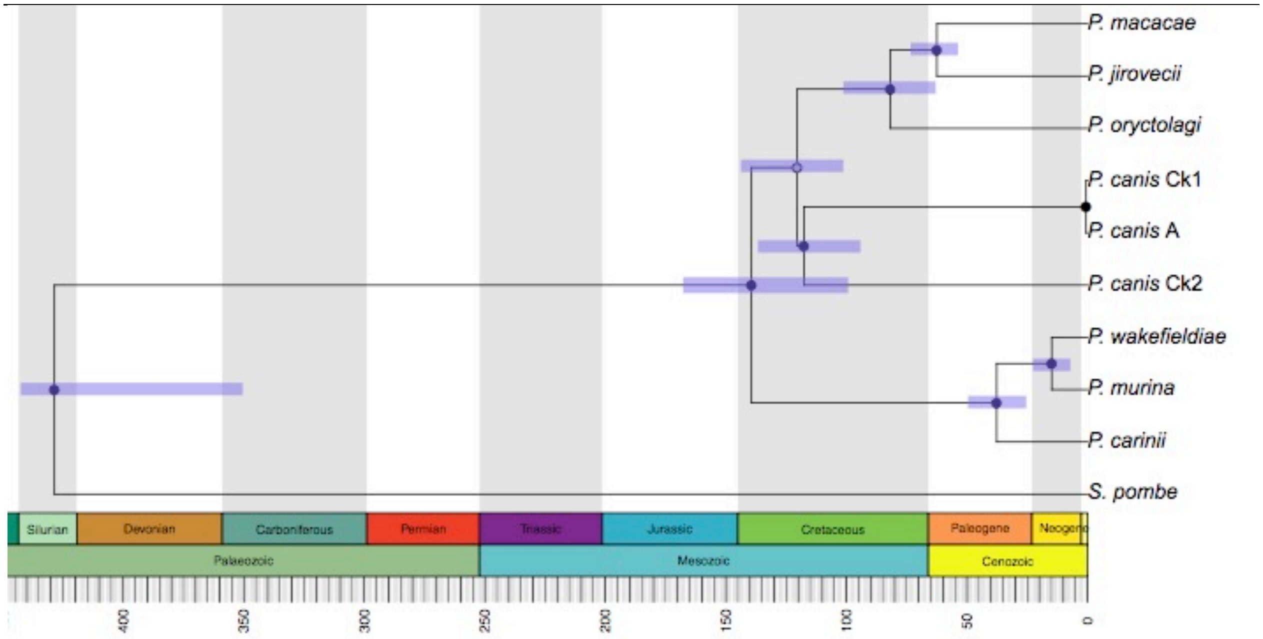

Figure S2. Maximum likelihood phylogeny constructed using a concatenated dataset of 15 genes from 33 *Pneumocystis* mitochondrial genomes including 7 *Pneumocystis* species. Pj, *P. jirovecii*; Pmac, *P.macacae*; Po, *P. oryctolagi*; Pcan, *P. canis*; Pcar, *P. carinii*; Pmur, *P. murina*; Pwk, *P. wakefieldiae*. The following genes were analyzed: cytochrome c oxidase subunits (*cox1*, *cox2, cox3*), ATP synthase F0 subunits (*atp6*, *atp8*, *atp9*), NADH dehydrogenase subunits (*nad1*, *nad2*, *nad3*, *nad5* and *nad6*), apocytochrome b (*cob*), ribonuclease P RNA (*rnpB*) and large and small subunit ribosomal RNAs (*rnl* and *rns*). Numbers in parentheses on the branches are the Shimodaira-Hasegawa [SH]-approximate likelihood ratio test (%) followed by the ultrafast bootstrap support (%).

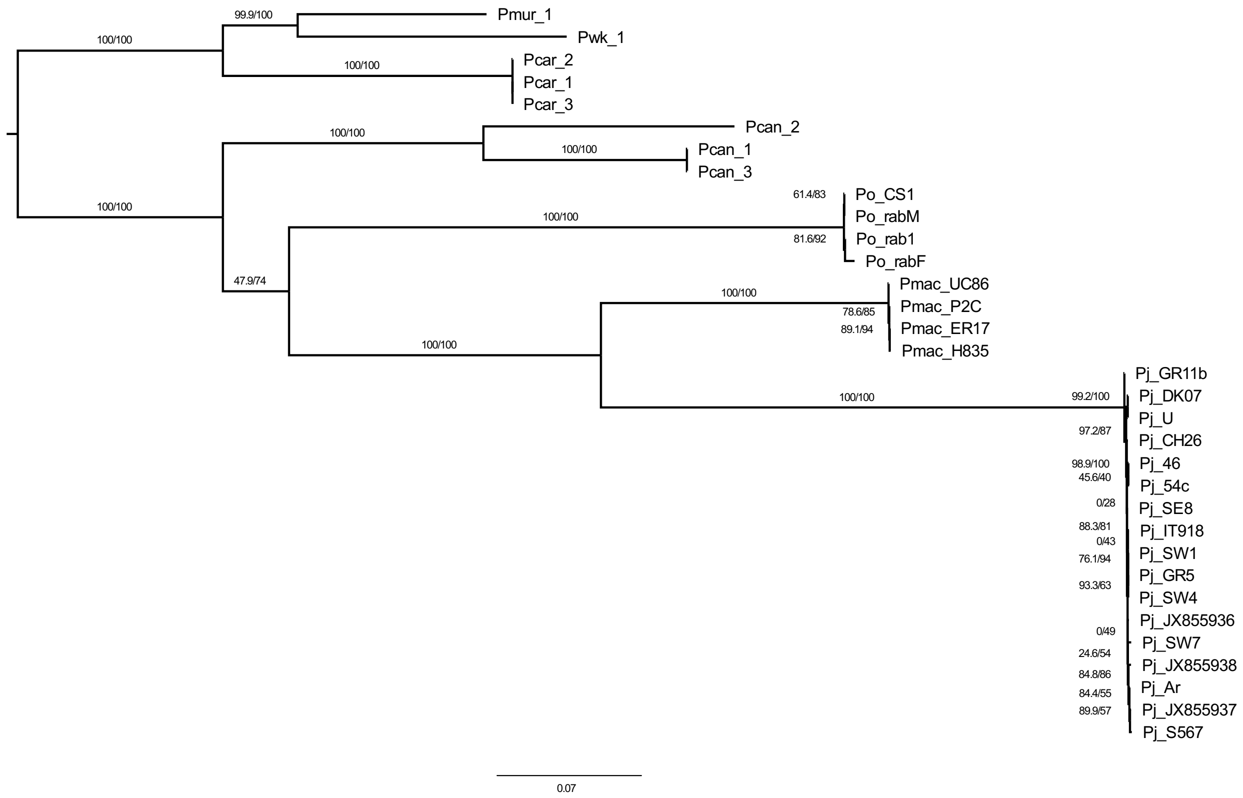

Figure S3. Genome-wide scans for footprints of natural selection in *Pneumocystis*. a, SNPs-based hierarchical clustering of *Pneumocystis* specimens (n = 59). b, Relative population divergence fixation indexes (F_{ST}) comparing *P. jirovecii* population to *P. macacae* and *P. oryctolagi*, respectively. c, Biplot of F_{ST} values comparing *P. jirovecii* population to *P. macacae* and *P. oryctolagi* populations colored according nucleotide diversities, showing that highly differentiated genomic regions tend to have a lower genetic diversity.

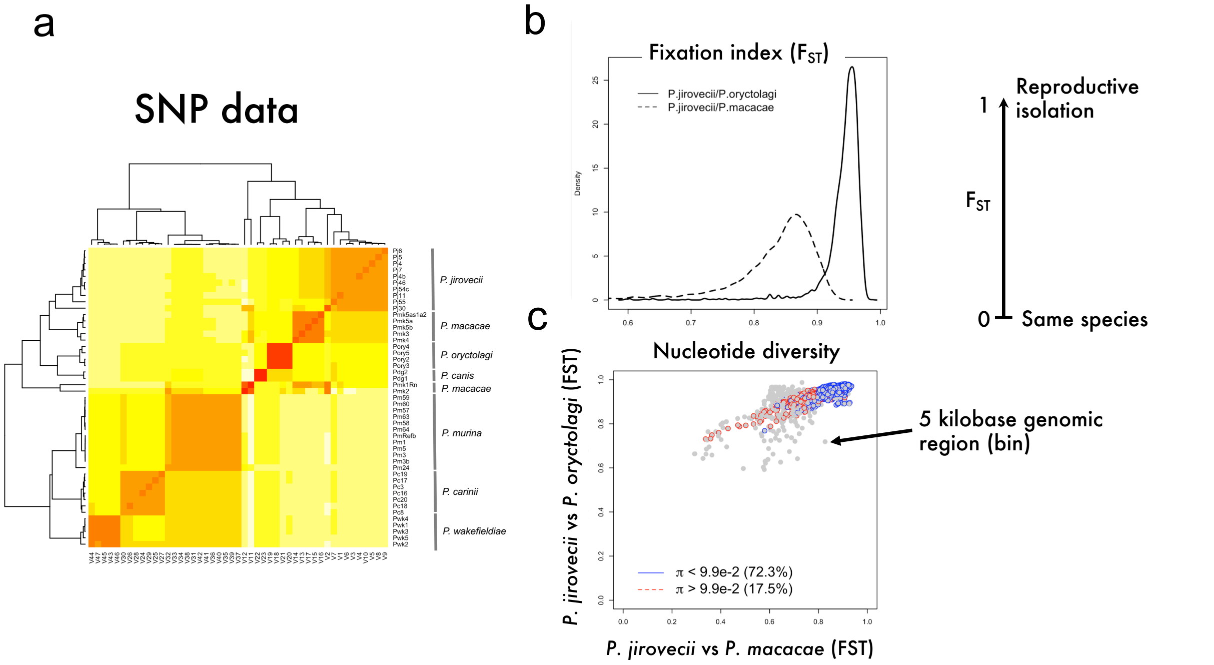

Figure S4. Evidence of ancient gene flow in rodent *Pneumocystis* only. Rooted phylogenetic network of seven *Pneumocystis* species inferred by PhyloNet based on 1,718 one-to-one ortholog gene trees. Reticulations are shown as blue lines with inheritance probability of 64%.

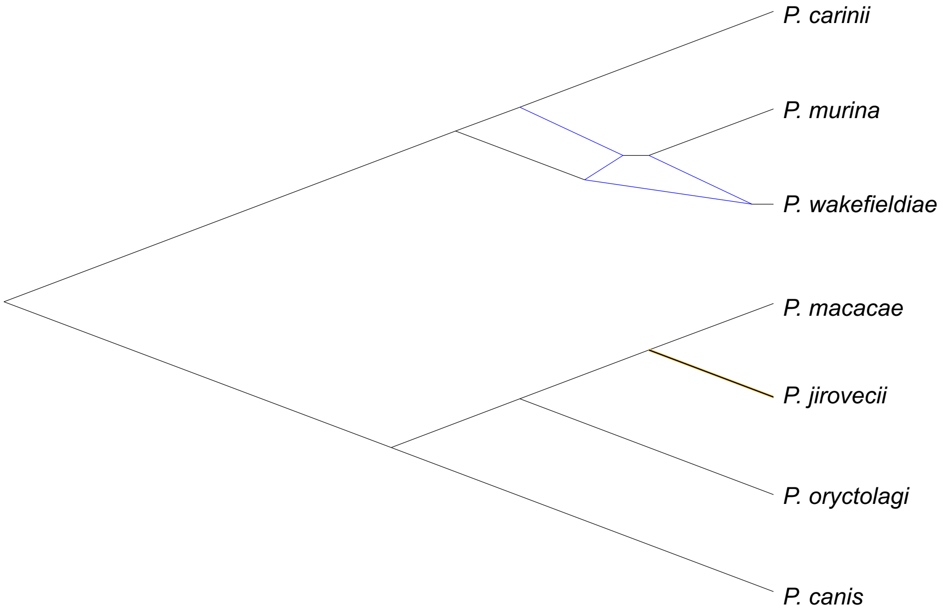

Figure S5. Evolution of arginine-glycine (RG) rich proteins in *P. macacae*. a, Sequence logos showing the frequency of amino acid composition in RG proteins. b, RNA-Seq of 1,518 out of 3,427 *P. macacae* genes with at least 10 Transcripts Per Kilobase Million (TPM) counts. The plot shows high gene expression levels of RG genes (n = 88/191; shown as light blue-colored points) relative to other genes (red). X-axis shows gene identifiers and y-axis shows the log2 transformed TPM counts. c, Analysis of a subset of *P. macacae* RGs (n = 6) using TOPALi based on the Difference of Sums of Squares method (DSS). Recombination is significantly detected at positions 75 to 150 of the alignment. Maximum likelihood of RG proteins. The blue star indicates the only protein with a signal peptide. d, Phylogenetic network of subtelomeric RG proteins showing reticulation events (possible recombination).

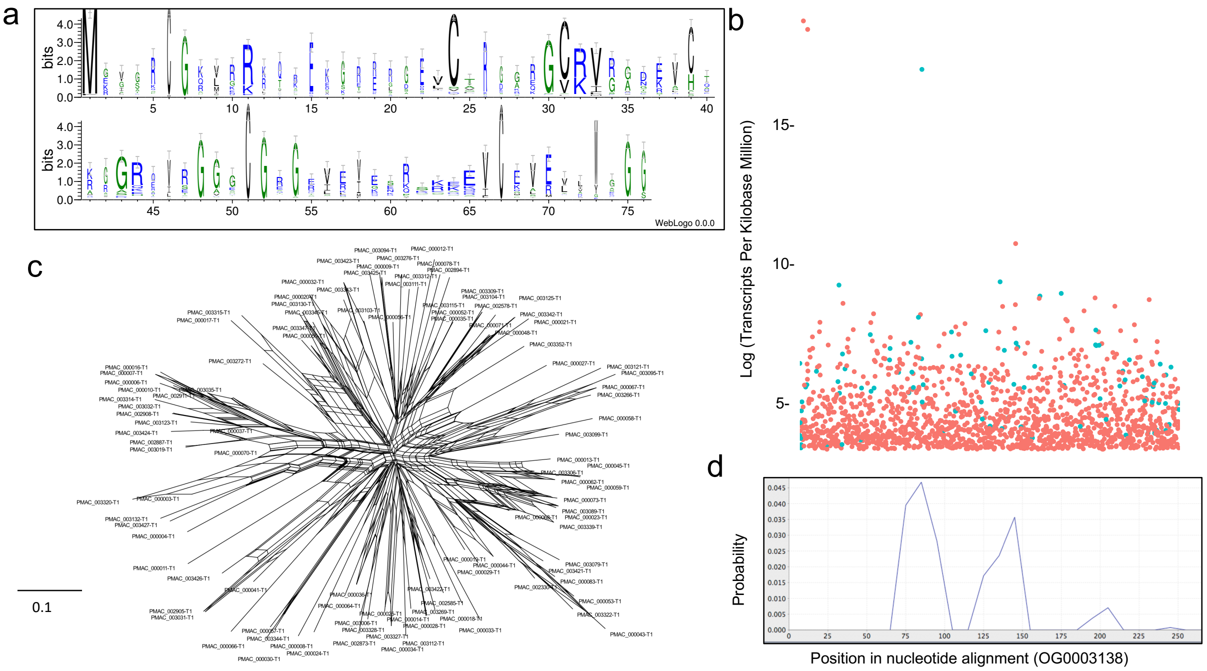

Figure S6. Heatmap showing gene family distribution in *Pneumocystis* species and related fungi. Binary profiles of 7,720 gene families. Filled black squares denote presence of at least one member of the family in the genome or absence (empty) across the gene catalogs of seven *Pneumocystis* species, 11 related fungi from the Taphrinomycotina subphylum and five fungi from different subphyla. Taphrinomycotina fungi are presented in boxes at the bottom. TPOP, *Taphrina populina*. TW, *T. wiesneri*. TFLA, *T. flavoruba*. TDEF, *T. deformans*. SJAP, *Schizosaccharomyces japonicus*. SPOM, *S. pombe*. SCRYO, *Schizosaccharomyces cryophilus*. SOCT, *S. octosporus*. Prab, *Pneumocystis oryctolagi*. Pmac, *P. macacae*. PcanAus, *P. canis* strain A. PcanCk, *P. canis* strain Ck1. Pwk, *P. wakefieldiae*. PjRu7, *P. jirovecii* strain *Ru7*. PcB80, *P. carinii* strain B80. Pmur, *P. murina*. RHIOR, *Rhizopus oryzae*. Bd, *Batrachochytrium dendrobatidis*. NIRR, *Neolecta irregularis*. PROTO, *Protomyces inouyei*. CNEO, *Cryptococcus neoformans*. NCRA, *Neurospora crassa*. SAICO, *Saitoella complicata*.

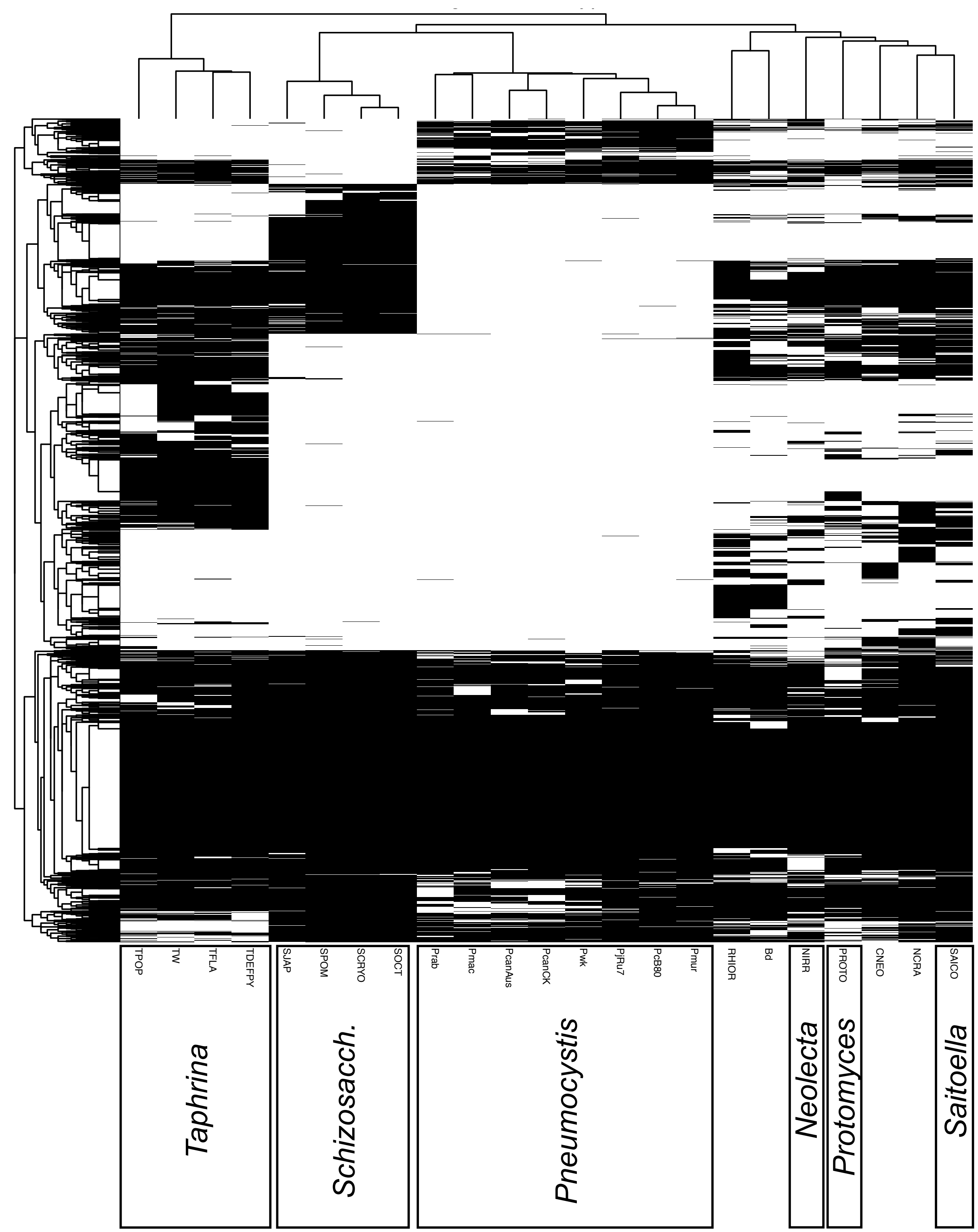
TPOP
TW
TFLA
TDEFPY
SJAP
SPOM
SCRYO
SOCT
Prab
Pmac
PcanAus
PcanCK
Pwk
PjRu7
PcB80
Pmur
RHIOR
Bd
NIRR
PROTO
CNEO
NCRA
SAICO
Taphrina
Schizosacch.
Pneumocystis
Neolecta
Protomyces
Saitoella

Figure S7. Expansion of kexin peptidase families in *Pneumocystis*. Maximum likelihood phylogenetic tree of all kexin genes with hits to Pfam domain PF00082. A total of 13 kexin genes are present in the *P. carinii* genome excluding 27 located in unassembled short contigs. All other species have only one copy except *P. wakefieldiae* which has three. The presence of a predicted GPI anchor and signal peptide is indicated by '+'. The number of predicted transmembrane regions is presented for each sequence. Sequences are labeled by a unique sequence identifier preceded by one of the species acronyms: PC, *P. carinii*; PWK, *P. wakefieldiae*; PM, *P. murina*; PCANCK, *P. canis* Ck1; PORYCT, *P. oryctolagi*; PJ, *P. jirovecii*; PMAC, *P. macacae*; SOCT, *Schizosaccharomyces octosporus*; Scryo, *S. cryophilus*; Spom, *S. pombe*; Sjap, *S. japonicus*.

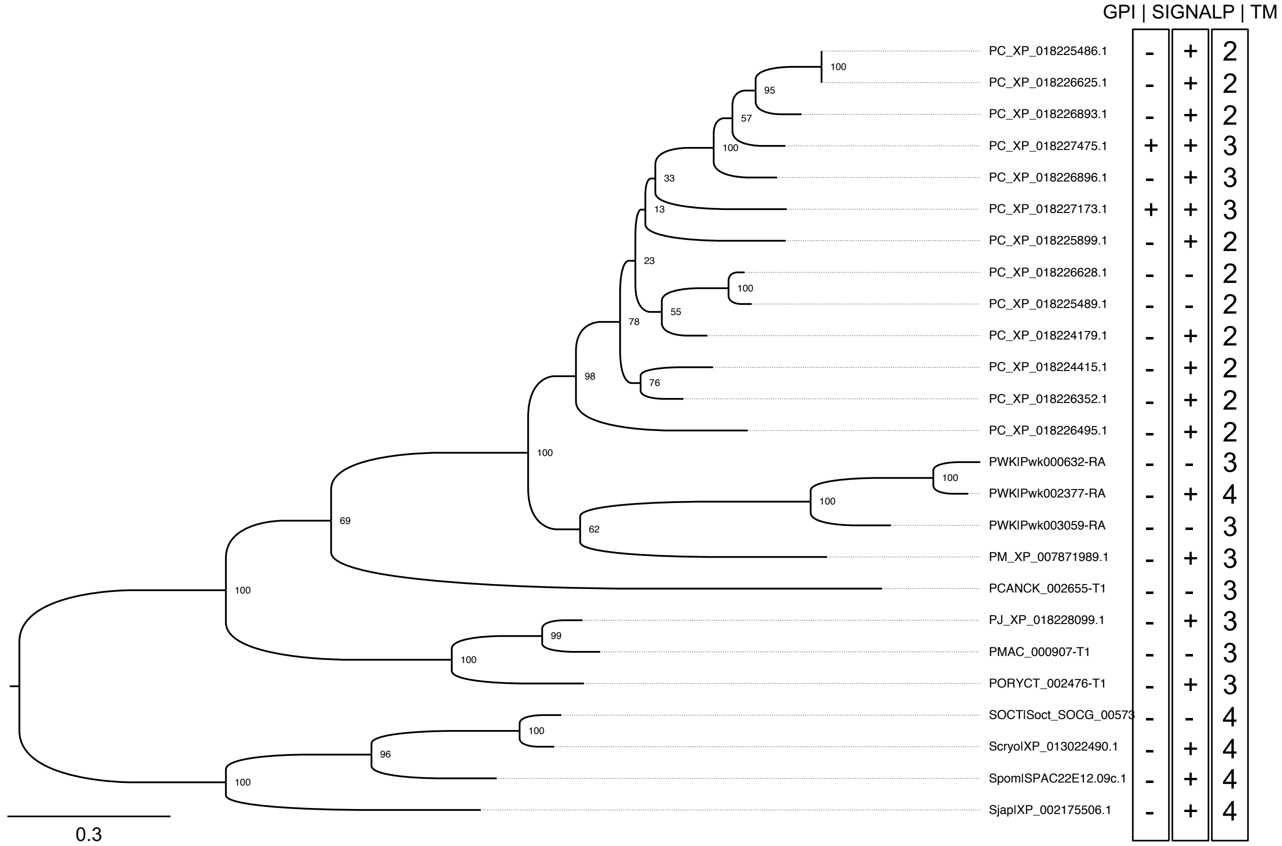

Figure S8. Evolutionary history of CFEM domains in *Pneumocystis* and other Taphrinomycotina. **a** Maximum likelihood phylogeny of CFEM domain-containing genes. **b** Reconciliation of domain and /species trees showing CFEM domain evolutionary history. Sequences are labeled by a unique sequence identifier preceded by one of the species acronyms: PJIR, *P. jirovecii*; PMAC, *P. macacae*; PORY, *P. oryctolagi*; PCAN, *P. canis* strain Ck1; PC, *P. carinii*; PMUR, *P. murina*; PWK, *P. wakefieldiae*. **c** Sequence logo showing the frequency of amino acid composition in *Pneumocystis* CFEM domains.

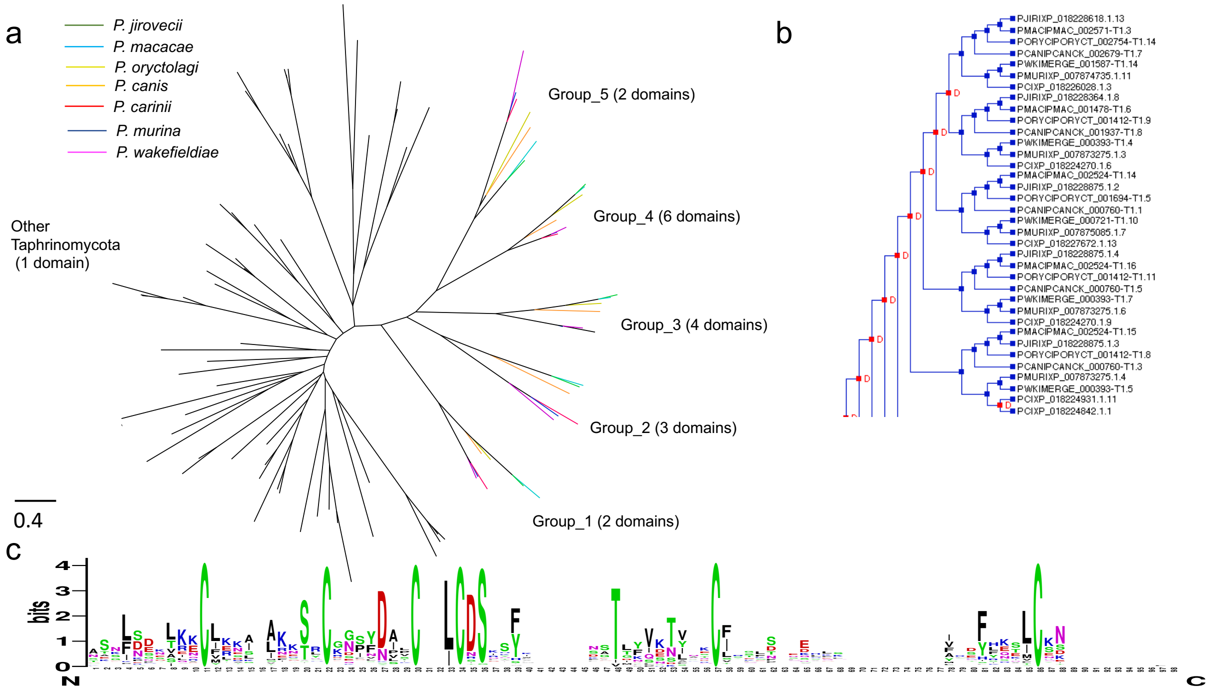

Figure S9. Evolutionary history of introns in *Pneumocystis* and Taphrinomycotina fungi. **a** Graphical display of a representative protein alignment showing the gaps and intron position with respect to each of the aligned sequence. Nongap positions are indicated by grey boxes. Intron positions are indicated by small colored tags that have one, two, or three overlapping discs, indicating intron phase. Conserved (i.e., unambiguously aligned) intron-bearing sites are shown by dotted vertical lines. **b** Bar chart of intron counts per species. **c** Prediction of ancestral intron densities. The graphical display shows the inferred intron counts at the inner nodes and terminal taxa. The purple discs at the nodes have proportional diameters to the inferred densities. On each branch, the inferred number of intron losses (negative numbers) and gains (positive numbers) are presented. The orange and green bars have proportional heights to loss and gain amounts, respectively. Species are labelled with the following acronyms: *P. jirovecii* (pjir), *P. macacae* (pmac), *P. oryctolagi* (pory), *P. canis* (pcan), *P. carinii* (pcar), *P. murina* (pmur), *P. wakefieldiae* (pwk), *Schizosaccharomyces pombe* (spom), *Neolecta irregularis* (nirr), *Taphrina deformans* (tdef).

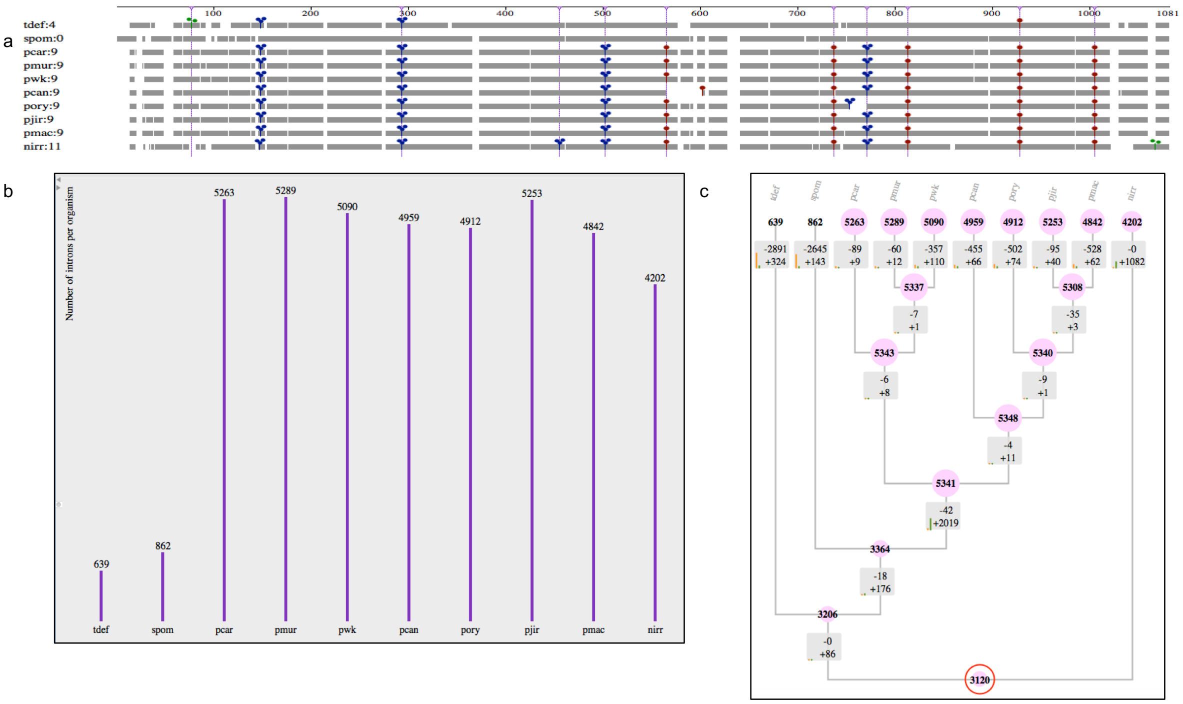
a
tdef:4
spom:0
pcar:9
pmur:9
pwk:9
pcan:9
pory:9
pjir:9
pmac:9
nirr:11
100
200
300
400
500
600
700
800
900
1000
1081
b
Number of introns per organism
639
862
5263
5289
5090
4959
4912
5253
4842
4202
tdef
spom
pcar
pmur
pwk
pcan
pory
pjir
pmac
nirr
c
tdef
spom
pcar
pmur
pwk
pcan
pory
pjir
pmac
nirr
639
862
5263
5289
5090
4959
4912
5253
4842
4202
-2891 +324
-2645 +143
-89 +9
-60 +12
-357 +110
-455 +66
-502 +74
-95 +40
-528 +62
-0 +1082
5337
-7 +1
5308
-35 +3
5343
-6 +8
5340
-9 +1
5348
-4 +11
5341
-42 +2019
3364
-18 +176
3206
-0 +86
3120

Figure S10. RAxML phylogeny and phylogenic networks of Major surface glycoprotein (msg) genes. **a** RAxML phylogeny of 482 Msg protein. **b** Phylogenetic network of *P. jirovecii* and *P. macacae msg* genes. The letters represent Msg families (A to E) and subfamilies (A1 to A3). **c** Phylogenetic network of *P. carinii*, *P. murina* and *P. wakefieldiae msg* genes. Note that the full phylogenetic network was divided in two parts (b and c) to ease visualization. Complete phylogenetic network data are available at DOI: 10.5281/zenodo.4450766.

Figure S10a.

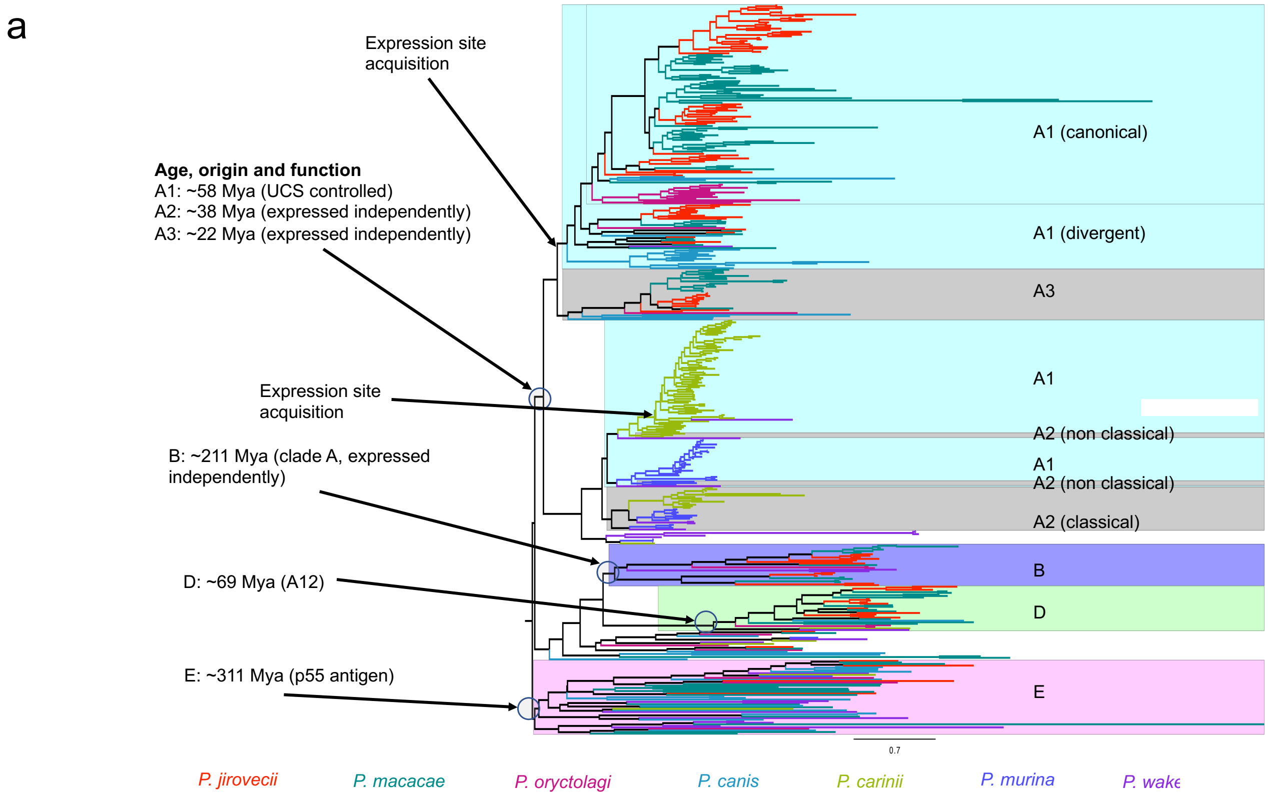

Figure S10b.

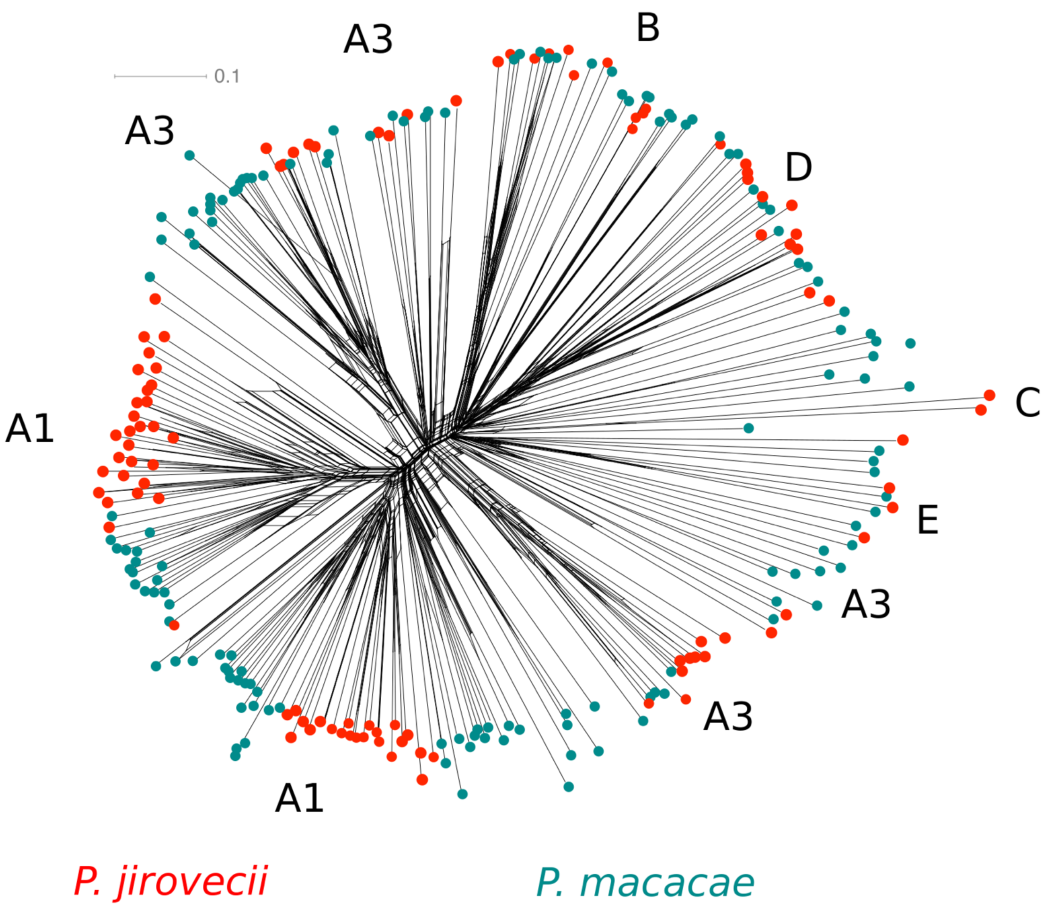

Figure S10c.

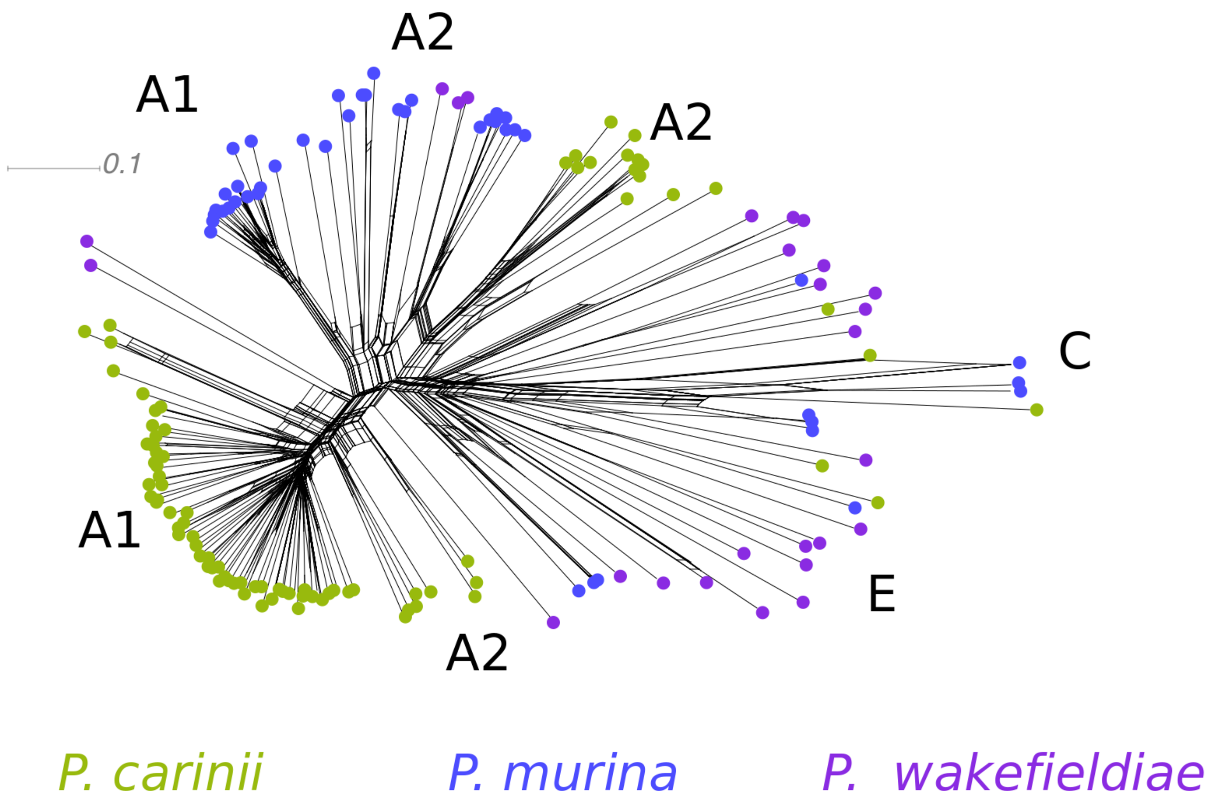

Figure S11. Phylodating of major surface glycoproteins in *Pneumocystis* and species divergence (*n* = 14 intervals of evolutionary ages analyzed). Horizontal lines represent the 95% confidence interval (CI) error bars of Bayesian estimates and the dotted vertical line represents the emergence of the *Pneumocystis* genus.

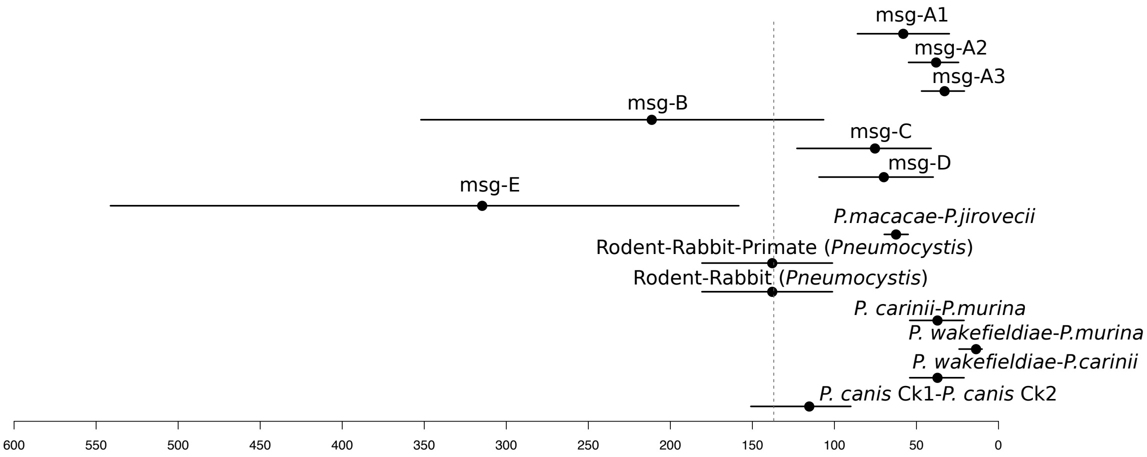

Supplementary Table 1. Clinical and demographic data of individual samples used in this study [a].

Species	Sample	Host species/strain	Collection dates (month, year)	Type of respiratory sample	Immune status/Condition	Host sex	Host age	Geographical location
P. jirovecii								
	Pj46	*Homo sapiens*	n.a	BAL	n.a	n.a	n.a	Bethesda, Maryland, USA
	Pj54c	*Homo sapiens*	06/2018	BAL	HIV infection	F	n.a	Bethesda, Maryland, USA
	Pj55	*Homo sapiens*	11/2018	BAL	Kidney transplantation	F	27	Chongqing, China
P. macacae								
	H835	*Macaca mulatta*	04/2013	Autopsy lungs	Experimentally infected with SIV	n.a	n.a	Bethesda, Maryland, USA
	P2C	*Macaca mulatta*	06/2018	Autopsy lungs	Experimentally infected with SIV	M	6 years	Bethesda, Maryland, USA
	CJ36	*Macaca mulatta*/Indian	06/2017	Autopsy lungs	Experimentally infected with SIV	F	n.a	Tulane National Primate Research Center, Covington, LA
	ER17	*Macaca mulatta*/Indian	12/2011	FFPE sections	Experimentally infected with SIV	M	8 years	Tulane National Primate Research Center, Covington, LA
	UC86	*Macaca mulatta*/Chinese	02/1997	FFPE sections	Experimentally infected with SIV	M	1 year	CNPRC, Davis, CA
	GL92	*Macaca mulatta*/Indian	01/2016	FFPE sections	Experimentally infected with SIV	M	9 years	Tulane National Primate Research Center, Covington, LA
P. oryctolagi								
	RABM	*Oryctolagus cuniculus* /NZW/ Il2rg KO	08/2017	Lungs	SCID	M	5 months	Ann Arbor, MI, Michigan
	RABF	*Oryctolagus cuniculus*	n.a/2005	Lungs	Spontaneous PCP at weaning	n.a	n.a	Tours, France
	RAB1	*Oryctolagus cuniculus*	n.a/2005	Lungs	IBC	n.a	n.a	Lille, France
	RAB2	*Oryctolagus cuniculus*	n.a/2005	Lungs	IBC	n.a	n.a	Lille, France
P. canis								
	Ck	*Canis lupus familiaris* /Cavalier King Charles Spaniel	10/1994	FFPE sections	Suspected immunodeficiency	M	1 year	Helsinki, Finland
	A	*Canis lupus familiaris* /Whippet mixed	02/2013	Lungs	Leukocytosis and neutrophilia	M	3 years	Vetmeduni, Austria
P. wakefieldiae/P. carinii								
	Pw2A	*Rattus norvegicus*/Long Evans rat	09/1995	Lungs	IBC	n.a	n.a	Cincinnati, OH, USA
	PwC1	*Rattus norvegicus*/Brown Norway rat	10/2000	Lungs	IBC	M	13 weeks	Cincinnati, OH, USA
	Pw1A	*Rattus norvegicus*/Brown Norway rat	10/1996	Lungs	IBC	n.a	n.a	Cincinnati, OH, USA
	Pw3A	*Rattus norvegicus*/Brown Norway rat	09/2002	Lungs	IBC	M	13 weeks	Cincinnati, OH, USA
	PwRN	*Rattus norvegicus*	10/1992	Lungs	IBC	n.a	n.a	Cincinnati, OH, USA
P. murina								
	D3A	CD40L KO mice/A549 cells	07/2018	Lungs	*In vitro* culture	n.a	3 days	Bethesda, Maryland, USA

Abbreviations: BAL, bronchoalveolar lavage; FFPE, formalin-fixed paraffin-embedded tissues; IBC, immunosuppressed by corticosteroid administration; PCP, *Pneumocystis* pneumonia; NZW/Il2rg KO, interleukin-2 receptor-ɣ knock out mutant rabbits; SCID, severe combined immunodeficiency; SIV, Simian immunodeficiency virus;
n.a., not available.

[a] Public raw sequencing data used in this study are presented separately in Supplementary Table 2.

Supplementary Table 2. Statistics and a posteriori classification of reads used in this study.

Species	*n*	Sample ID	Type	Host ID	Raw paired reads (millions)	*Pneumocystis* reads (%)	Median coverage	Data source	NCBI BioProject/SRA accession #
P. jirovecii									
	1	Pj46	DNA	46	26.6	0.1	4.8	This study	SRR12304674
	2	Pj54c	DNA	54c	46.2	0.8	47.4	This study	SRR12304306
	3	Pj55	DNA	55	24.6	6.8	10.1	This study	SRR12300271
	4	RU817b [a c]	DNA	RU	10.7	65.0	85.4	This study	n.a.
	5	RU7[a c]	DNA	RU	70.5	47.0	342.3	Ma *et al.* [28]	SRR1043749
	6	RU12 [a c]	DNA	RU	70.6	48.0	353.3	Ma *et al.* [28]	SRR1043747
	7	RU817 [a c]	DNA	RU	70.6	46.0	338.7	Ma *et al.* [28]	SRR1043750
	8	SE8 [c]	DNA	SE8	95.5	15.0	1,364.5	Cissé *et al.* [10]	PRJEB2702
	9	Z [c]	DNA	Z	89.6	9.4	11.6	Ma *et al.* [28]	SRR5822089, SRR5825481, SRR5825487
	10	W [c]	DNA	W	91.8	17.0	64.6	Ma et *al.* [28]	SRR5822088
	11	RN1	RNA	RN1	21.6	5.3	11.8	Cissé *et al.* [10]	PRJEB3063
	12	RN2	RNA	RN2	25.5	0.6	6.7	Cissé *et al.* [31]	SRR5822079
P. macacae									
	1	M2 [c]	DNA	H835	7.7	1.1	4.6	Cissé *et al.* [31]	SRR12323870
	2	MP3 [c]	DNA	H835	3.7	0.3	1.5	Cissé *et al.* [31]	SRR12323870
	3	H835	DNA	H835	0.6	1.0	1.9	Cissé *et al.* [31]	SRR12323870
	5	CJ36	DNA	CJ36	35.4	<0.1	5.4	This study	SRR12323878
	6	ER17	DNA	ER17	52.2	0.5	8.7	This study	PRJNA648108
	7	UC86	DNA	UC86	38.0	4.4	8.1	This study	PRJNA648112
	8	P2C [bc]	DNA	P2C	61.7	68.2	1,394.4	This study	SRR11785744
	9	S1A2 [bc]	DNA	P2C	24.0	19.3	151.8	This study	SRR11785744
	10	P2C [d]	DNA	P2C	1.6	5.0	n.a	This study	SRR11785744

	11	GL92	DNA	GL92	29.8	2.1	4.5	This study	PRJNA648115
	12	RN1	RNA	H835	18.2	1.9	13.4	Cissé et al. 2018 [31]	SRR5822119
	13	RN2	RNA	P2C	22.0	91.3	12.0	This study	SRR11785742
P. oryctolagi									
	1	RABF	DNA	RABF	73.0	3.6	9.6	This study	SRR11789048
	2	RABM [c]	DNA	RABM	73.5	4.4	11.1	This study	SRR11789047
	3	RAB1	DNA	RAB1	17.7	1.0	9.2	This study	SRR11789046
	4	RAB2	DNA	RAB2	20.7	1.1	25.4	This study	SRR11789045
P. canis									
	1	Ck	DNA	Ck	111.4	1.9	17.1	This study	SRR11795436
	2	A	DNA	A	38.8	5.1	15.3	This study	SRR11908556
P. wakefieldiae									
	1	2A [c]	DNA	Pw2A	23.2	17.6	167.4	This study	SRR11794283
	2	C1 [c]	DNA	PwC1	29.5	0.5	2.0	This study	SRR11794286
	3	1A [c]	DNA	Pw1A	67.5	79.7	2,193.2	This study	SRR11794285
	4	3A [c]	DNA	Pw3A	66.3	33.2	899.2	This study	SRR11794284
	5	2A [c]	RNA	RNA	39.7	25.2	n.a	This study	SRR11794282
P. carinii									
	1	B80 [c]	DNA	B80	28.8	56.8	389.6	Ma *et al.* [28]	SRR1043726, SRR1043727
	2	B50 [c]	DNA	B50	11.7	41.4	116.8	Ma *et al.* [28]	SRR1043724
	3	SE6 [c]	DNA	SE6	28.6	21.3	53.6	Cissé *et al.* [10]	ERR047567
	4	Pc1 [c]	RNA	70954	14.3	4.9	160.0	Ma *et al.* [28]	SRR2156857
	5	Pc2 [c]	RNA	70955	13.7	6.3	188.4	Ma *et al.* [28]	SRR2156986
	6	Pc3 [c]	RNA	70956	13.5	5.6	162.2	Ma *et al.* [28]	SRR2156987
	7	2A [c]	DNA	Pc2A	23.2	0.7	6.4	This study	SRR11794283
	8	C1 [c]	DNA	PcC1	29.5	7.5	87.1	This study	SRR11794286

	9	1A [c]	DNA	Pc1A	67.5	0.4	10.9	This study	SRR11794285
	10	3A [c]	DNA	Pc3A	66.3	3.8	98.4	This study	SRR11794284
	11	2A [c]	RNA	Pc2A	39.7	26.1	n.a	This study	SRR11794282
P. murina									
	1	Pm1 [c]	DNA	C1	17.6	23.8	107.1	Ma *et al.* [28]	SRR6001186
	2	Da3 [c]	DNA	Da3	23.2	4.8	24.1	Ma *et al.* [28]	SRR6001196
	3	Da1 [c]	DNA	Da1	19.3	3.8	15.2	Ma *et al.* [28]	SRR6001188
	4	MS96 [c]	DNA	MS96	19.9	9.8	46.5	Ma *et al.* [28]	SRR6001185
	5	A123 [c]	DNA	A123	18.9	29.2	139.9	Ma *et al.* [28]	SRR6001197
	6	Pm24	RNA	A549_PC	20.8	4.8	27.5	This study	SRR12313714
	7	Pm57 [e]	RNA	Pm57	48.9	19.2	4.2	Cushion *et al.* [32]	SRR6721590
	8	Pm58 [e]	RNA	Pm58	43.6	18.3	5.8	Cushion *et al.* [32]	SRR6721591
	9	Pm59 [e]	RNA	Pm59	49.	19.4	3.8	Cushion *et al.* [32]	SRR6721592
	10	Pm60 [e]	RNA	Pm60	42.3	14.4	4.6	Cushion *et al.* [32]	SRR6721593
	11	Pm63 [e]	RNA	Pm63	44.3	11.6	4.6	Cushion *et al.* [32]	SRR6721596
	12	Pm64 [e]	RNA	Pm64	46.6	19.1	5.6	Cushion *et al.* [32]	SRR6721597

The variability in the percentages of *Pneumocystis* reads among samples depends on a variety of factors, including the type of sample, the level of infection in an individual host, and the DNA enrichment and extraction methods utilized.

[a] All RU# samples were aliquots of the same cell suspension from a small piece of lungs of the same patient. Since these data were used for the *P. jirovecii* strain RU7 reference genome, they were excluded for population genetics or intra strain comparison.
[b] Samples P2C and S1A2 are DNA extracts from the same animal P2C (Table 1).
[c] Samples were enriched for *Pneumocystis* DNA prior to sequencing. The samples P2C (no. 8) and S1A2 (no.9) were enzymatically treated to deplete macaque DNA. All *P. wakefieldiae* samples went through gradient centrifugation and filtration.
[d] Sequenced using Oxford Nanopore sequencing platform.
[e] Single end Illumina reads. Only healthy controls were used from this study.

Supplementary Table 3. Statistics of different *Pneumocystis* genome assemblies.

		Species								
	Features	P. macacae	P. oryctolagi	P. canis Ck1	P. canis Ck2	P. canis A	P. wakefieldiae	P. jirovecii	P. carinii	P. murina
**Genomic DNA**										
	Source	This study	This study	This study	This study	This study	This study	Ma et al. [28]	Ma et al. [28]	Ma et al. [28]
	Host	Macaque	Rabbit	Dog Ck	Dog Ck	Dog A	Rat	Human	Rat	Mouse
	Strain	P2C	CS1	Ck1[a]	Ck2 [a]	A	2A	RU7	B80	B123
	DNA amplification	None	WGA	None	None	None	None	None	None	None
**Scaffolds**										
	Sequencing technology	Nanopore + Illumina	Illumina	Illumina	Illumina	Illumina	Illumina	PacBio + Illumina	PacBio + Illumina	PacBio + Illumina
	Total length (Mb)	8.2	7.6	7.9	3.6	7.4	7.3	8.3	7.6	7.4
	Number	16[b]	38	78	315	33	17	20[b]	17[b]	17[b]
	Coverage	1,394.4	13.8	17.2	n.a	15.3	2663.2	342.3	389.6	139.9
	N50 (kb)	505.3	534.6	457.1	174.4	422.4	480.6	454.5	465.1	491.3
	GC content (%)	29.1	28.6	26.3	29.7	25.9	29.8	28.7	27.8	26.9
	With telomeric motif (n)	7	2	2	4	0	5	7	6	9
	Karyotype (n)	n.a	14 [33]	n.a	n.a	n.a	14 [11]	17-19 [34]	13-15 [35]	17 [36]
**Nuclear genome assembly completeness (%)**										
	CEGMA (%)	89.9	89.9	91.1	14.9	93.1	93.1	91.1	91.3	91.9
	BUSCO (%)	86.2	92.4	90.7	15.6	91.8	92.4	95.1	94.8	94.4
	FGMP (%)	92.2	89.6	93.4	49.2	93.4	92.6	93.1	94.4	92.2
**Nuclear annotation statistics**										
	Protein-coding genes (n)[c]	3,427	2,961	3,476	2,135	3,077	3,221	3,765	3,646	3,838
	Ribosomal RNA genes (n)	5	10	5	5	5	5	5	5	5
	Transfer RNA genes (n)	47	47	46	18	47	47	46	45	47
	Exons (n)	21,608	21,574	21,632	8,544	20,216	23,126	21,770	21,808	22,085
	Exons per gene (n)	6	6	6	5	6	7	6	6	6
	Orthogroups (n)	3,280	3,102	3,395	1,296	3,060	3,133	3,643	3,602	3,574
	Orphans (n)	190	204	80	44	17	25	120	42	50
	Msg (n)	106	9[c]	22	36 [c]	23	17	161	93	61
	Repeats (%) [d]	2.6	2.8	2.7	1.6	2.7	1.8	2.7	3.0	2.4
**Mitochondrial genome statistics**										
	Source	This study	This study	This study	This study	This study	This study	Ma et al. [37]	Ma et al.[37]	Ma et al. [37]
	Total length (bp)	21,232	24,512	21,750	21,413	21,368	23,752	35,626	26,119	24,608
	Scaffolds or contigs (n)	1	1	1	1	1	1	1	1	1
	Predicted topology	Circular	Linear	Linear	Linear	Linear	Linear	Circular	Linear	Linear
	GC content (%)	28.9	31.5	29.3	29.4	29.4	30.0	25.7	29.8	29.8
	Protein-coding genes (n)[c]	15	15	15	15	15	15	15	15	15
	rRNA genes (n)	3	3	3	3	3	3	3	3	3
	tRNA genes (n)	22	22	22	23	23	22	25	25	28

[a] *P. canis* genome assemblies Ck1 and Ck2 are from the same dog Ck.
[b] Excluding short contigs containing *msg* or kexin genes.
[c] Possibly incomplete due to difficulties in assembling full-length *msg* genes from Illumina reads alone.
[d] DNA transposons, retrotransposons and simple low complexity AT rich repeats.

[e] Including one hypothetic protein-coding gene (orf195).
Abbreviations: WGA: Whole Genome Amplification; CEGMA, Core Eukaryotic Genes Mapping Approach; BUSCO, Benchmarking Universal Single-Copy Orthologs; FGMP, Fungal Genome Mapping Pipeline; n.a., not available.

Supplementary Table 4. Genome rearrangements among different *Pneumocystis* species.

GR	GO	Syntenic blocks (n)[a]	Inversions (n)[b]	Genome rearrangement breakpoints[c]						
				Total refined (n)	Genic (n)	IGS	Repeats[d]	Divergence (%)	Telomeric	Msg
P. jirovecii	*P. macacae*	43	23	29	13	16	19	15.7	2	0
P. macacae	*P. jirovecii*	43	23	33	25	8	11		0	0
P. jirovecii	*P. oryctolagi*	225	26	142	97	45	31	20.5	1	0
P. oryctolagi	*P. jirovecii*	225	26	132	84	48	24		4	0
P. jirovecii	*P. canis* Ck1	168	48	82	42	40	31	21.3	2	0
P. canis Ck1	*P. jirovecii*	168	48	79	40	39	20		1	0
P. jirovecii	*P. carinii*	134	53	82	44	38	34	22.6	2	0
P. carinii	*P. jirovecii*	134	53	85	70	15	19		2	0
P. jirovecii	*P. murina*	112	45	79	39	40	35	22.4	2	0
P. murina	*P. jirovecii*	112	45	82	60	21	10		0	0
P. jirovecii	*P. wakefieldiae*	128	46	86	46	40	38	22.9	1	0
P. wakefieldiae	*P. jirovecii*	128	46	89	58	31	10		0	0
P. wakefieldiae	*P. murina*	29	9	10	0	10	1	12.0	0	0
P. murina	*P. wakefieldiae*	29	9	10	5	5	1		0	0
P. wakefieldiae	*P. carinii*	44	14	20	13	7	4	14.9	1	0
P. carinii	*P. wakefieldiae*	44	14	20	13	7	4		1	0
P. carinii	*P. murina*	29	14	10	3	7	2	12.3	0	0
P. murina	*P. carinii*	29	14	10	0	10	0		0	0
S. octoporus	*S. cryophilus*	71	19	30	18	12	1	20.9	0	NA
S. cryophilus	*S. octoporus*	71	19	24	17	7	2		0	NA
S. pombe	*S. japonicus*	708	NA	160	159	1	8	21.0	0	NA
S. japonicus	*S. pombe*	708	NA	160	154	6	0		0	NA

Abbreviations: BPs: breakpoints; IGS: intergenic spaces; GR: reference genome; GO: queried genome; Msg: major surface glycoprotein encoding regions; N.A, not applicable. [a] Syntenic blocks defined by MAUVE/GRIMM; [b] Inversions were inferred using MAUVE;[c] BPs were refined using Cassis and identified in the GR (reference genome) not in the GO; [d] Repeats as identified using RepeatMasker. These elements might overlap with genic and IGS regions.

Supplementary Table 5. Pairwise nucleotide divergence (%) among *Pneumocystis* genomes.

Species (Specimen)	*P. jirovecii* (RU7)	*P. jirovecii* (SE8)	*P. jirovecii* (SE2178)	*P. macacae* (P2C)	*P. oryctolagi* (CS1)	*P. canis* (Ck1)	*P. canis* (Ck2)	*P. canis* (A)	*P carinii* (B80)	*P carinii* (Ccin)	*P. carinii* (SE6)	*P. murina* (B123)	*P. wakefieldiae* (2A)	Data source	NCBI accession #
P. jirovecii (RU7)	0.0													Ma et al. [28]	GCA_001477535.1
P. jirovecii (SE8)	0.2	0.0												Cisse et al.[10]	GCA_000333975.2
P. jirovecii (SE2178)	0.8	0.5	0.0											Schmid et al. [38]	GCA_002571455.1
P. macacae (P2C)	15.7	15.1	15.6	0.0										This study	JABMLO000000000
P. oryctolagi (CS1)	20.5	20.4	20.5	20.8	0.0									This study	JABTEG000000000
P. canis (Ck1)	21.3	21.2	21.2	21.5	21.6	0.0								This study	JABTEF000000000
P. canis (Ck2)	20.6	20.6	20.6	20.7	20.7	15.5	0.0							This study	JABVCJ000000000
P. canis (A)	21.3	21.2	21.3	21.5	21.6	0.5	15.5	0.0						This study	JACEFK000000000
P. carinii (B80)	22.6	22.5	22.6	22.8	22.8	22.5	21.0	22.5	0.0					Ma et al. [28]	GCA_001477545.1
P. carinii (Ccin)	23.1	23.0	23.1	22.8	23.2	23.1	21.0	23.1	5.0	0.0				Slaven et al. [9]	This study
P. carinii (SE6)	21.9	21.8	21.8	22.0	22.1	21.8	20.6	21.8	1.4	4.2	0.0			Cisse et al.[10]	This study
P. murina (B123)	22.4	22.3	22.4	22.5	22.6	22.3	21.0	22.3	12.3	14.7	12.5	0.0		Ma et al. [28]	GCA_000349005.2
P. wakefieldiae (2A)	22.9	22.8	22.8	23.0	23.1	22.8	21.2	22.8	14.9	16.5	14.9	12.0	0.0	This study	PRJNA632570

Consensus genome assemblies for additional low coverage *P. jirovecii* (*n* = 4) and *P. macacae* (*n* = 5) were generated. Pairwise divergence estimates are available at https://github.com/ocisse/pneumocystis_evolution/blob/master/docs/inter_intra_div/scores.csv.

Supplementary references

1 Langmead, B. & Salzberg, S. L. Fast gapped-read alignment with Bowtie 2. *Nat Methods* **9**, 357-359, doi:10.1038/nmeth.1923 (2012).

2 Vurture, G. W. *et al.* GenomeScope: fast reference-free genome profiling from short reads. *Bioinformatics* **33**, 2202-2204, doi:10.1093/bioinformatics/btx153 (2017).

3 Bankevich, A. *et al.* SPAdes: a new genome assembly algorithm and its applications to single-cell sequencing. *J Comput Biol* **19**, 455-477, doi:10.1089/cmb.2012.0021 (2012).

4 Kent, W. J. BLAT--the BLAST-like alignment tool. *Genome Res* **12**, 656-664, doi:10.1101/gr.229202 (2002).

5 Altschul, S. F. *et al.* Gapped BLAST and PSI-BLAST: a new generation of protein database search programs. *Nucleic Acids Res* **25**, 3389-3402 (1997).

6 Bolger, A. M., Lohse, M. & Usadel, B. Trimmomatic: a flexible trimmer for Illumina sequence data. *Bioinformatics* **30**, 2114-2120, doi:10.1093/bioinformatics/btu170 (2014).

7 Stanke, M., Schoffmann, O., Morgenstern, B. & Waack, S. Gene prediction in eukaryotes with a generalized hidden Markov model that uses hints from external sources. *BMC Bioinformatics* **7**, 62, doi:10.1186/1471-2105-7-62 (2006).

8 Hauser, P. M. *et al.* Comparative Genomics Suggests that the Fungal Pathogen Pneumocystis Is an Obligate Parasite Scavenging Amino Acids from Its Host's Lungs. *Plos One* **5**, doi:ARTN e15152

10.1371/journal.pone.0015152 (2010).

9 Slaven, B. E. *et al.* Draft assembly and annotation of the Pneumocystis carinii genome. *J Eukaryot Microbiol* **53 Suppl 1**, S89-91, doi:10.1111/j.1550-7408.2006.00184.x (2006).

10 Cisse, O. H., Pagni, M. & Hauser, P. M. De novo assembly of the Pneumocystis jirovecii genome from a single bronchoalveolar lavage fluid specimen from a patient. *MBio* **4**, e00428-00412, doi:10.1128/mBio.00428-12 (2012).

11 Cushion, M. T., Keely, S. P. & Stringer, J. R. Molecular and phenotypic description of Pneumocystis wakefieldiae sp. nov., a new species in rats. *Mycologia* **96**, 429-438 (2004).

12 Rice, P., Longden, I. & Bleasby, A. EMBOSS: the European Molecular Biology Open Software Suite. *Trends Genet* **16**, 276-277 (2000).

13 Edgar, R. C. MUSCLE: multiple sequence alignment with high accuracy and high throughput. *Nucleic Acids Res* **32**, 1792-1797, doi:10.1093/nar/gkh340 (2004).

14 Stamatakis, A. RAxML version 8: a tool for phylogenetic analysis and post-analysis of large phylogenies. *Bioinformatics* **30**, 1312-1313, doi:10.1093/bioinformatics/btu033 (2014).

15 Morgulis, A. *et al.* Database indexing for production MegaBLAST searches. *Bioinformatics* **24**, 1757-1764, doi:10.1093/bioinformatics/btn322 (2008).

16 Pryszcz, L. P. & Gabaldon, T. Redundans: an assembly pipeline for highly heterozygous genomes. *Nucleic Acids Res* **44**, e113, doi:10.1093/nar/gkw294 (2016).

17 Grabherr, M. G. *et al.* Genome-wide synteny through highly sensitive sequence alignment: Satsuma. *Bioinformatics* **26**, 1145-1151, doi:10.1093/bioinformatics/btq102 (2010).

18 English, K., Peters, S. E., Maskell, D. J. & Collins, M. E. DNA analysis of Pneumocystis infecting a Cavalier King Charles spaniel. *J Eukaryot Microbiol* **Suppl**, 106S (2001).

19 Song, J. *et al.* Bacterial and Pneumocystis Infections in the Lungs of Gene-Knockout Rabbits with Severe Combined Immunodeficiency. *Front Immunol* **9**, 429, doi:10.3389/fimmu.2018.00429 (2018).

20 Dei-Cas, E. *et al.* Pneumocystis oryctolagi sp. nov., an uncultured fungus causing pneumonia in rabbits at weaning: review of current knowledge, and description of a new taxon on genotypic, phylogenetic and phenotypic bases. *FEMS Microbiol Rev* **30**, 853-871, doi:10.1111/j.1574-6976.2006.00037.x (2006).

21 Lowe, T. M. & Eddy, S. R. tRNAscan-SE: a program for improved detection of transfer RNA genes in genomic sequence. *Nucleic Acids Res* **25**, 955-964, doi:10.1093/nar/25.5.955 (1997).

22 Kielbasa, S. M., Wan, R., Sato, K., Horton, P. & Frith, M. C. Adaptive seeds tame genomic sequence comparison. *Genome Res* **21**, 487-493, doi:10.1101/gr.113985.110 (2011).

23 Korneliussen, T. S., Albrechtsen, A. & Nielsen, R. ANGSD: Analysis of Next Generation Sequencing Data. *BMC Bioinformatics* **15**, 356, doi:10.1186/s12859-014-0356-4 (2014).

24 Ma, L., Cisse, O. H. & Kovacs, J. A. A Molecular Window into the Biology and Epidemiology of Pneumocystis spp. *Clin Microbiol Rev* **31**, e00009-00018, doi:10.1128/CMR.00009-18 (2018).

25 Gournas, C., Prevost, M., Krammer, E. M. & Andre, B. Function and Regulation of Fungal Amino Acid Transporters: Insights from Predicted Structure. *Adv Exp Med Biol* **892**, 69-106, doi:10.1007/978-3-319-25304-6_4 (2016).

26 Lipschik, G. Y., Masur, H. & Kovacs, J. A. Polyamine metabolism in Pneumocystis carinii. *J Infect Dis* **163**, 1121-1127, doi:10.1093/infdis/163.5.1121 (1991).

27 Velasco, I., Tenreiro, S., Calderon, I. L. & Andre, B. Saccharomyces cerevisiae Aqr1 is an internal-membrane transporter involved in excretion of amino acids. *Eukaryot Cell* **3**, 1492-1503, doi:10.1128/EC.3.6.1492-1503.2004 (2004).

28 Ma, L. *et al.* Genome analysis of three Pneumocystis species reveals adaptation mechanisms to life exclusively in mammalian hosts. *Nat Commun* **7**, 10740, doi:10.1038/ncomms10740 (2016).

29 Oltmanns, O. & Bacher, A. Biosynthesis of riboflavine in Saccharomyces cerevisiae: the role of genes rib 1 and rib 7. *J Bacteriol* **110**, 818-822 (1972).

30 Tanaka, T., Tateno, Y. & Gojobori, T. Evolution of vitamin B6 (pyridoxine) metabolism by gain and loss of genes. *Mol Biol Evol* **22**, 243-250, doi:10.1093/molbev/msi011 (2005).

31 Cisse, O. H. *et al.* Comparative Population Genomics Analysis of the Mammalian Fungal Pathogen Pneumocystis. *MBio* **9**, e00381-00318, doi:10.1128/mBio.00381-18 (2018).

32 Cushion, M. T. *et al.* Gene Expression of Pneumocystis murina after Treatment with Anidulafungin Results in Strong Signals for Sexual Reproduction, Cell Wall Integrity, and Cell Cycle Arrest, Indicating a Requirement for Ascus Formation for Proliferation. *Antimicrob Agents Chemother* **62**, doi:10.1128/AAC.02513-17 (2018).

33 Cho SR, P. Y., Moon HN, Lee SH, Hong ST. Karyotypes of Pneumocystis carinii derived from several mammals. *Korean J Parasitol* **37**, 271–275 (1999).

34 Lundgren, B., Cotton, R., Lundgren, J. D., Edman, J. C. & Kovacs, J. A. Identification of Pneumocystis carinii chromosomes and mapping of five genes. *Infect Immun* **58**, 1705-1710 (1990).

35 Hong, S. T. *et al.* Karyotypes of Pneumocystis carinii from Korean rats. *Kisaengchunghak Chapchi* **30**, 183-189, doi:10.3347/kjp.1992.30.3.183 (1992).

36 Keely, S. P., Fischer, J. M., Cushion, M. T. & Stringer, J. R. Phylogenetic identification of Pneumocystis murina sp. nov., a new species in laboratory mice. *Microbiology* **150**, 1153-1165, doi:10.1099/mic.0.26921-0 (2004).

37 Ma, L. *et al.* Sequencing and characterization of the complete mitochondrial genomes of three Pneumocystis species provide new insights into divergence between human and rodent Pneumocystis. *Faseb J* **27**, 1962-1972, doi:10.1096/fj.12-224444 (2013).

38 Schmid-Siegert, E. *et al.* Mechanisms of Surface Antigenic Variation in the Human Pathogenic Fungus Pneumocystis jirovecii. *MBio* **8**, e01470-01417, doi:10.1128/mBio.01470-17 (2017).
