# Supplementary material for: Genomic insights into the host specific adaptation of the Pneumocystis genus
Source: Commun Biol. 2021 Mar 8;4:305. doi: 10.1038/s42003-021-01799-7 (PMC7940399; doi:10.1038/s42003-021-01799-7)
Supplement: Supplementary file 2 — Description of Additional Supplementary Files [file 42003_2021_1799_MOESM2_ESM.pdf]

Description of Additional Supplementary Files

File name: Supplementary Data 1

Description: Annotation of Pneumocystis macacae genome subtelomeric regions.

Pneumocystis jirovecii genome-wide signatures of selection

File name: Supplementary Data 2

Description: Pneumocystis jirovecii genome-wide signatures of selection.
