# Supplementary material for: Genomic insights into the host specific adaptation of the Pneumocystis genus
Source: Commun Biol. 2021 Mar 8;4:305. doi: 10.1038/s42003-021-01799-7 (PMC7940399; doi:10.1038/s42003-021-01799-7)
Supplement: Supplementary file 5 — Reporting Summary [file 42003_2021_1799_MOESM5_ESM.pdf]

nature research

Corresponding author(s): Ousmane H. Cisse

Last updated by author(s): 1/18/2021

Reporting Summary

Nature Research wishes to improve the reproducibility of the work that we publish. This form provides structure for consistency and transparency in reporting. For further information on Nature Research policies, see our Editorial Policies and the Editorial Policy Checklist.

Statistics

For all statistical analyses, confirm that the following items are present in the figure legend, table legend, main text, or Methods section.

n/a	Confirmed	
☐	☒	The exact sample size (n) for each experimental group/condition, given as a discrete number and unit of measurement
☐	☒	A statement on whether measurements were taken from distinct samples or whether the same sample was measured repeatedly
☐	☒	The statistical test(s) used AND whether they are one- or two-sided<br>*Only common tests should be described solely by name; describe more complex techniques in the Methods section.*
☒	☐	A description of all covariates tested
☐	☒	A description of any assumptions or corrections, such as tests of normality and adjustment for multiple comparisons
☐	☒	A full description of the statistical parameters including central tendency (e.g. means) or other basic estimates (e.g. regression coefficient) AND variation (e.g. standard deviation) or associated estimates of uncertainty (e.g. confidence intervals)
☐	☒	For null hypothesis testing, the test statistic (e.g. F, t, r) with confidence intervals, effect sizes, degrees of freedom and P value noted<br>*Give P values as exact values whenever suitable.*
☐	☒	For Bayesian analysis, information on the choice of priors and Markov chain Monte Carlo settings
☐	☒	For hierarchical and complex designs, identification of the appropriate level for tests and full reporting of outcomes
☒	☐	Estimates of effect sizes (e.g. Cohen's d, Pearson's r), indicating how they were calculated

Our web collection on statistics for biologists contains articles on many of the points above.

Software and code

Policy information about availability of computer code

Data collection	no software was used
Data analysis	De novo assembly analyses were performed using Spades (https://github.com/ablab/spades). Quantification of transcript abundance was performed using Kallisto. Genome annotation was performed using funannotate (https://github.com/nextgenusfs/funannotate). Phylogenomic analyses were performed using BLAST, RAXML , IQTREE and BEAST2. All custom bioinformatic analyses were conducted using Perl v5.26.0 (http://www.perl.org/) or Python v.3.6 (http://www.python.org) scripts. Pipelines were written using Snakemake. Custom scripts and pipelines are available at https://github.com/ocisse/pneumocystis_evolution. Statistical analyses were conducted in R.

For manuscripts utilizing custom algorithms or software that are central to the research but not yet described in published literature, software must be made available to editors and reviewers. We strongly encourage code deposition in a community repository (e.g. GitHub). See the Nature Research guidelines for submitting code & software for further information.

Data

Policy information about availability of data

All manuscripts must include a data availability statement. This statement should provide the following information, where applicable:

- Accession codes, unique identifiers, or web links for publicly available datasets
- A list of figures that have associated raw data
- A description of any restrictions on data availability

The datasets generated during and/or analyzed during the current study are available in the NCBI BioProject (https://www.ncbi.nlm.nih.gov/bioproject/): Pneumocystis macacae strain P2C (no. PRJNA632025); P. oryctolagi strain CS1 (PRJNA632560); P. canis strain Ck1 (PRJNA632556); P. canis strain Ck2 (PRJNA632878); P. canis strain A (PRJNA636786);P. wakefieldiae strain 2A (PRJNA632570); P. jirovecii strain 55 (PRJNA647920), P. jirovecii strain 54c (PRJNA648092), P. jirovecii strain 46 (PRJNA648096), P. macacae strain CJ36 (PRJNA648103), P. macacae strain ER17 (PRJNA648108), P. macacae strain UC86 (PRJNA648112) and P.

macacae strain GL92 (PRJNA648115).

Field-specific reporting

Please select the one below that is the best fit for your research. If you are not sure, read the appropriate sections before making your selection.

- [x] Life sciences
- [ ] Behavioural & social sciences
- [ ] Ecological, evolutionary & environmental sciences

For a reference copy of the document with all sections, see nature.com/documents/nr-reporting-summary-flat.pdf

Life sciences study design

All studies must disclose on these points even when the disclosure is negative.

Sample size	No sample-size calculation was performed. Pneumocystis infected samples are in short supply and usually contain less 0.1% of Pneumocystis DNA. Therefore all samples were processed to recover enough DNA for genomic studies.
Data exclusions	No data were excluded from the analyses
Replication	Findings were not replicated because the DNA/RNA extraction methods used are destructive, and only limited amounts of biological specimens are available.
Randomization	Not relevant for this study
Blinding	Not relevant for this study

Reporting for specific materials, systems and methods

We require information from authors about some types of materials, experimental systems and methods used in many studies. Here, indicate whether each material, system or method listed is relevant to your study. If you are not sure if a list item applies to your research, read the appropriate section before selecting a response.

Materials & experimental systems

n/a	Involved in the study	
x		Antibodies
x		Eukaryotic cell lines
x		Palaeontology and archaeology
	x	Animals and other organisms
	x	Human research participants
x		Clinical data
x		Dual use research of concern

Methods

n/a	Involved in the study	
x		ChIP-seq
x		Flow cytometry
x		MRI-based neuroimaging

Animals and other organisms

Policy information about studies involving animals; ARRIVE guidelines recommended for reporting animal research

Laboratory animals	The supplementary table 1 contains all the clinical information and demographic data of individual samples used in this study
Wild animals	The study did not involve wild animals
Field-collected samples	Three P. jirovecii samples were obtained as bronchoalveolar lavage from patients at the NIH Clinical Center in Bethesda, MD, USA and Chongqing Medical University in Chongqing, China.<br>Six P. macacae samples were obtained as frozen lung tissues or formalin fixed paraffin embedded (FFPE) tissue sections prepared from SIV-infected rhesus macaques at the NIH Animal Center, Bethesda, Maryland (n = 2), the Tulane National Primate Research Center, Covington, Louisiana (n = 3), and the UC Davis California National Primate Research Center, Davis, California, USA (n = 1).<br>Four P. oryctolagi samples were obtained as frozen lung tissues from one rabbit with severe combined immunodeficiency at the University of Michigan, Ann Arbor, Michigan, USA, or as DNA from two corticosteroid treated rabbits and one rabbit with spontaneous Pneumocystis infection at the Institut Pasteur de Lille and the Institut National de la Recherche Agronomique de Tours Pathologie Aviaire et Parasitologie, Tours, France.<br>P. canis samples were obtained as DNA from one Cavalier King Charles Spaniel dog at the University of Helsinki, Finland and one Whippet mixed-breed at the University of Veterinary Medicine, Vienna, Austria.<br>P. murina organisms were obtained from heavily-infected CD40L-KO mice following a short-term in vitro culture. Genomic data obtained from P. murina isolates were combined with previously sequenced public data (Supplementary Table 2) and used for population genomics analysis (section "Speciation history of the Pneumocystis genus" and Supplementary Note 1).<br>One frozen cell pellet and 4 agarose gel blocks containing P. wakefieldiae and P. carinii were obtained from immunosuppressed rats (one gel block per rat) housed at the Cincinnati VA Medical Center, Veterinary Medicine Unit, Cincinnati, Ohio.

Ethics oversight	Animal and human subject experimentation guidelines of the National Institutes of Health (NIH) were followed in the conduct of this study. Studies of human and mouse Pneumocystis infection were approved by NIH Institutional Review Board (IRB) protocols 99-I-0084 and CCM 19-05, respectively. The collection and processing of a single P. jirovecii human bronchoalveolar lavage sample from China (Pj55) was approved by the IRB of the First Affiliated Hospital of Chongqing Medical University, China (protocol no. 20172901). Written informed consent was obtained from the patient for the participation in this study. The authors confirmed that personal identity information of the patient data was unidentifiable from this report. The National Institute of Allergy and Infectious Diseases (NIAID) Division of Intramural Research Animal Care and Use Program, as part of the NIH Intramural Research Program, approved all experimental procedures pertaining to the macaques (protocol LVD 26). Nonhuman primate study protocols were approved by the Institutional Animal Care and Use Committee of the University of California, Davis (protocol no. 7092), the Tulane National Primate Research Center (TNPRC) and the Institutional Animal Care and Use Committee (IACUC) (protocol no. P0351R). Studies of rabbit Pneumocystis infection were reviewed and approved by the Institutional Animal Care and Use Committee of the University of Michigan (protocol no. RO00008218). For rabbit samples obtained France, the conditions for care of laboratory animals stipulated in European guidelines were followed (See: Council directives on the protection of animals for experimental and other scientific purposes, and J. Off. Communautés Européennes, 86/609/EEC, 18 December 1986, L358). Samples from Pneumocystis infected dog were collected as diagnostic samples and approved for only for research purpose. The owner's consents for using samples and data were obtained on admission of the case and no further ethics permission was required because it was a routine diagnostic case and did not qualify as an animal experiment. Studies of rat Pneumocystis infection were approved by the Veteran Affairs animal protocol (VA ACORP #17-12-05-01). Clinical information and demographic data of the groups of individuals are presented in Supplementary Table 1.

Note that full information on the approval of the study protocol must also be provided in the manuscript.

Human research participants

Policy information about studies involving human research participants

Population characteristics	Immunocompromised individuals
Recruitment	The collection and processing of a single P. jirovecii human bronchoalveolar lavage sample from China (Pj55)
Ethics oversight	Animal and human subject experimentation guidelines of the National Institutes of Health (NIH) were followed in the conduct of this study. The collection and processing of a single P. jirovecii human bronchoalveolar lavage sample from China (Pj55) was approved by the IRB of the First Affiliated Hospital of Chongqing Medical University, China (protocol no. 20172901). Written informed consent was obtained from the patient for the participation in this study. The authors confirmed that personal identity information of the patient data was unidentifiable from this report.

Note that full information on the approval of the study protocol must also be provided in the manuscript.
